# Multi-channel PINN: investigating scalable and transferable neural networks for drug discovery

**DOI:** 10.1186/s13321-019-0368-1

**Published:** 2019-07-09

**Authors:** Munhwan Lee, Hyeyeon Kim, Hyunwhan Joe, Hong-Gee Kim

**Affiliations:** Biomedical Knowledge Engineering Laboratory, Seoul National University, 1 Gwanak-ro, Seoul, Republic of Korea

**Keywords:** Deep neural networks, Machine learning, Compound–protein interaction, Proteochemometrics, Cheminformatics

## Abstract

Analysis of compound–protein interactions (CPIs) has become a crucial prerequisite for drug discovery and drug repositioning. In vitro experiments are commonly used in identifying CPIs, but it is not feasible to discover the molecular and proteomic space only through experimental approaches. Machine learning’s advances in predicting CPIs have made significant contributions to drug discovery. Deep neural networks (DNNs), which have recently been applied to predict CPIs, performed better than other shallow classifiers. However, such techniques commonly require a considerable volume of dense data for each training target. Although the number of publicly available CPI data has grown rapidly, public data is still sparse and has a large number of measurement errors. In this paper, we propose a novel method, *Multi-channel PINN*, to fully utilize sparse data in terms of representation learning. With representation learning, *Multi-channel PINN* can utilize three approaches of DNNs which are a classifier, a feature extractor, and an end-to-end learner. *Multi-channel PINN* can be fed with both low and high levels of representations and incorporates each of them by utilizing all approaches within a single model. To fully utilize sparse public data, we additionally explore the potential of transferring representations from training tasks to test tasks. As a proof of concept, *Multi-channel PINN* was evaluated on fifteen combinations of feature pairs to investigate how they affect the performance in terms of highest performance, initial performance, and convergence speed. The experimental results obtained indicate that the multi-channel models using protein features performed better than single-channel models or multi-channel models using compound features. Therefore, *Multi-channel PINN* can be advantageous when used with appropriate representations. Additionally, we pretrained models on a training task then finetuned them on a test task to figure out whether *Multi-channel PINN* can capture general representations for compounds and proteins. We found that there were significant differences in performance between pretrained models and non-pretrained models.

**Electronic supplementary material:**

The online version of this article (10.1186/s13321-019-0368-1) contains supplementary material, which is available to authorized users.

## Introduction

Analysis of compound–protein interactions (CPIs) has become an important prerequisite for both discovering novel drugs for known protein targets and repurposing new targets for current drugs [1–3]. Exploring both molecular and proteomic space is a highly challenging and cost-intensive procedure. Each space is enormous and heterogeneous, moreover, most of the CPIs space remains to be discovered. For example, there are roughly $10^{8}$ synthesized compounds potentially developed into novel drugs [4, 5] but they are a small fraction of drug-like compounds, which the total is estimated on the order of between $10^{24}$ and $10^{60}$ [5, 6]. As for the targets of the compounds, there are about 200,000 reviewed human protein records [7]. In vitro experiments are commonly used in identifying CPIs, but it is not feasible to discover molecular and proteomic space only through experimental approaches. In silico models have emerged to aid traditional experiments by narrowing down the search space and prioritizing molecules with the highest potential [8–11].

Traditional in silico models can be grouped into two approaches, which are structure-based methods [12–14] and ligand-based methods [15–17]. In addition to the conventional approaches, proteochemometrics (PCM) methods have been proposed to predict CPIs by incorporating both ligand and target space within a single model [18–21]. First, structure-based methods yield reasonable prediction performance and visually interpretable results. Structure-based methods use three-dimensional (3D) simulation for molecular docking to discover CPIs. AutoDock [22], Glide [23], Fred [24], and AtomNet [25] are examples of docking tools. However, the methods have two major limitations: (1) intensive computational complexity and (2) the shortage of 3D structure data for compounds and proteins. Therefore, ligand-based and PCM methods are preferred in most cases.

Secondly, ligand-based methods depend on a basic assumption called the molecular similarity principle [26]. The assumption is that similar compounds are used to interact with similar proteins, where Quantitative Structure–Activity Relationship (QSAR) model is one of the representative examples. With the advent of machine learning (ML) algorithms, ligand-based methods, such as Naïve Bayes (NB) [27, 28], random forest (RF) [29], support vector machines (SVM) [30], deep neural networks (DNNs) [31] and multi-task neural networks [32, 33], have gained popularity. However, molecular activity alone is not sufficient to identify the bioactivity.

In contrast to ligand-based methods, PCM methods build a model using each compound and protein pair as the input data to fully utilize both proteomic and molecular space. Due to their pair-based modelling, PCM methods are able to predict the interactions between novel compounds and new proteins. PCM methods have recently demonstrated their performance in various tasks such as the identification of new drug combinations [34], prediction of interactions between drug and target [35], and CPIs prediction for G protein coupled receptor (GPCR) and protein kinase targets [36]. In addition, PCM has the potential to utilize information from various multispecies into a single model [18, 37]. Therefore, PCM methods have drawn attention in discovering CPI space [20].

DNN algorithms have recently been applied to predict CPI pairs and performed better than other shallow classifiers such as RF, NB and SVM [38, 39]. In addition to using basic DNNs called feedforward neural networks (FFNN), a previous study [40] has proposed pairwise input neural networks (PINN). As a variation of a FFNN, a PINN consists of two separated layers and one concatenated layer. Each separated layer is fed with a different feature as the input (i.e. compound and protein) and then each layer is concatenated before classifying the classes. Before the concatenated layers, each separated layer is independently composed without connection from other layers. This architecture allows PINN to reduce the total number of parameters in the networks by about 50% compared to the conventional FFNN without degradation in performance (see Additional file 1: Table S1). The architecture is also suitable for PCM methods, which utilize both compound and protein features. However, the majority of DNNs commonly require a considerable volume of data for each training target. Although the number of public available CPI pairs has grown rapidly, it is still not sufficient to model CPI space [41].

Moreover, as a recent study by Lenselink et al. [38] pointed out, public data can have a large number of errors due to the use of different scientific protocols. The authors presented a high-quality benchmark dataset and compared the performance between various combinations of descriptors, methods (PCM and QSAR), machine learning algorithms, and validation partitioning. The study found that PCM models generally exceed QSAR models under the same conditions. PCM-based DNNs algorithms outperformed the other models on both evaluation sets (temporal validation and random validation).

To complete the modelling of CPI space [20], there is still room for improvement for PCM-based DNNs in terms of representation learning. DNNs can be utilized with three approaches including a classifier, a feature extractor, and an end-to-end learner. As a classifier, DNN algorithms in drug discovery are generally fed with manually crafted features and predict the bioactivity. DNNs can also be used as a feature extractor for compound and protein descriptors [42, 43] to fully utilize the information in large-scale dataset such as ZINC for compounds [44] and UniProt for proteins [45]. As an end-to-end learner, DNNs can learn representations from raw data such as SMILES string of compounds and amino acid sequence of proteins. End-to-end learning manages the whole learning process from feature extraction to classification in a single model.

**Fig. 1:**
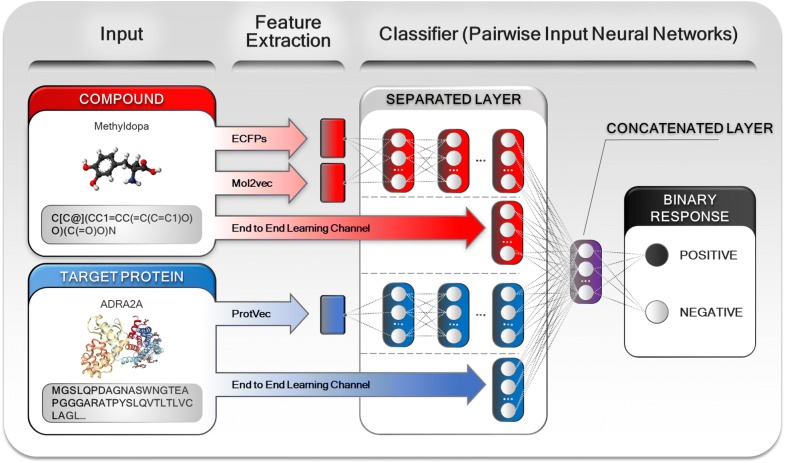
Schematic representations of *Multi-channel PINN* (*MCPINN*). *MCPINN* utilizes the three approaches of DNN in terms of a classifier, a feature extractor, and an end-to-end learner. *MCPINN* can incorporate both low and high level representations in a single model

In this paper, we propose a novel multi-channel PCM-based DNN called *Multi-channel PINN* (*MCPINN*). In order to make full use of sparse data, *MCPINN* utilizes three approaches of DNNs which are a classifier, a feature extractor, and an end-to-end learner. This model can be fed with both low and high levels of representations and can incorporate each of them into a single model (Fig. 1). With PINN architecture, this model takes both compounds and proteins into the input layer. It takes SMILES strings, ECFPs and vectors embedded by Mol2vec [42] for compounds and amino acid sequences and vectors embedded by ProtVec [43] for proteins. By incorporating the three approaches of DNNs, *MCPINN* can learn multiple representations to model the CPI data space. In addition to improving the model, we explore the potential ability of *MCPINN* to transfer the generalized representations from a high quality and well balanced training dataset to a strongly imbalanced test dataset.

As a proof of concept, we evaluated *MCPINN* on a standardized benchmark dataset [38] obtained from ChEMBL, using MCC and ROC as evaluation metrics. To investigate the effect of each feature, *MCPINN* was evaluated with six combinations of single-channel feature pairs. *MCPINN* was also evaluated with nine combinations of multi-channel feature pairs to explore the synergy effects of low and high levels of representations. The models were investigated in terms of not only highest performance but also initial performance and convergence speed. To test whether *MCPINN* can transfer general representations of compounds and proteins to a new task, we pretrained models on a training task, which is the benchmark dataset used above, and then finetuned the pretrained models on a test task Tox21 [46]. The transferability of *MCPINN* was evaluated in terms of initial performance, speed of convergence, and highest performance using two metrics for validation MCC and PRC. Therefore, this study contributes to “the complete modelling of CPI space” [20] by full use of representation ability of DNNs as a classifier, a feature extractor, and an end-to-end learner and additionally by transferring the generalized representations from training tasks to test task.

## Results and discussion

### Investigating the representation learning ability

The first part of this study focuses on the representation learning ability of *MCPINN*. To figure out the contribution of each feature on the predictive performance of the model, *MCPINN* was evaluated with fifteen combinations of feature pairs, which contained six pairs from single-channel features and nine pairs from multi-channel features. There are three features for compounds: SMILES, ECFP, and Mol2vec, where SMILES is a low-level representation. The performance based on the feature concatenated ECFP and Mol2vec was evaluated but omitted because the concatenated feature models did not provide improvement in performance compared to the Mol2vec or ECFP models separately (as can be seen in Additional file 1: Table S2). Two features are used for proteins: ProtVec and the amino acid sequence which is a low-level representation. For low-level representations (SMILES and amino acid sequence) Dilated CNN is applied as an end-to-end learner. Recurrent Neural Network models were also trained but omitted due to their poor performance, which can be seen in Additional file 1: Figures S1 and S2.

There are fifteen models based on the combinations of feature pairs and are listed in Table 1 with shortened names. The models were evaluated on a benchmark dataset [38] using two metrics for validation the Matthew Correlation Coefficient (MCC) and Receiver Operating Characteristic Area Under the Curve (ROC).

**Table 1:** The shortened names for combinations of features for *SCPINN* and *MCPINN*

Model name	Channel type	Compound feature	Protein feature
$SC_1$	Single-channel	SMILES	AA sequence
$SC_2$		SMILES	ProtVec
$SC_3$		Mol2vec	AA sequence
$SC_4$		Mol2vec	ProtVec
$SC_5$		ECFP	AA sequence
$SC_6$		ECFP	ProtVec
$MC_1$	Multi-channel for protein	SMILES	AA sequence and ProtVec
$MC_2$		Mol2vec	AA sequence and ProtVec
$MC_3$		ECFP	AA sequence and ProtVec
$MC_4$	Multi-channel for compound	SMILES and Mol2vec	AA sequence
$MC_5$		SMILES and Mol2vec	ProtVec
$MC_6$		SMILES and ECFP	AA sequence
$MC_7$		SMILES and ECFP	ProtVec
$MC_8$	Multi-channel for both features	SMILES and Mol2vec	AA sequence and ProtVec
$MC_9$		SMILES and ECFP	AA sequence and ProtVec

#### Comparison between single-channel models

**Fig. 2:**
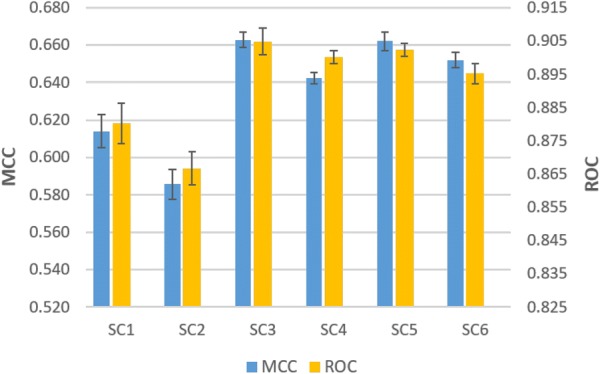
Comparison of predictive performance between *SCPINN*. On the left y-axis the MCC is shown, while on the right y-axis the ROC score is shown and error bars indicate SEM. Mean MCC is 0.636 (± 0.03) and mean ROC is 0.892 (± 0.02)

Above all, to investigate the effect of each feature on the predictive performance of the model, the six feature pairs are explored with Single-channel PINN (*SCPINN*). *SCPINN* is a basic model that is fed with only one feature for each protein and compound respectively as shown in Table 1. The prediction performance of each model is shown in Fig. 2. The average performance of *SCPINN* models was an MCC of 0.636 ± 0.03 and a ROC of 0.892 ± 0.02. Overall the differences in performances between the metrics scores were similar to each other. It is observed that the biggest difference in performance between the *SCPINN* models was the use of a high-level representation (ECFP and Mol2vec) in the chemical feature instead of a low-level representation (SMILES). For example, the average performance of the models using ECFP and Mol2vec for compounds was an MCC of 0.66 ± 0.008 and a ROC of 0.90 ± 0.004, while the average performance of the models using SMILES was an MCC of 0.60 ± 0.014 and a ROC of 0.87 ± 0.007.

On the other hand, the models using ProtVec did not outperform the models using amino acid sequence with Dilated CNN for the overall models, regardless of the types of chemical features used. The average MCC of models using amino acid sequence was 0.646 (± 0.023) and mean ROC was 0.896 (± 0.011), while the average MCC of models using ProtVec was 0.627 (± 0.029) and the mean ROC was 0.887 (± 0.015).

This difference in performance seems to be based on whether or not the feature extraction method is able to capture the order of the amino acid sequences in the feature vector, in addition to the content itself. The Dilated CNN model can featurize the entire sequence of a protein in terms of the order and content, whereas ProtVec has a limitation in that it does not reflect the order of the sequence in the feature vector. ProtVec divides the sequence into N-grams to make the word units, performs individual embedding on each N-gram word, and then sums up all the embedding vectors regardless of the orders. Therefore, different proteins could have the same embedding vectors with ProtVec, provided the same N-grams are used.

Secondly, there is room to improve operations in Mol2vec and ProtVec to prevent incorrect representations of embedded compounds and proteins. In Mol2vec and ProtVec, the sum operation reconstructs embedded word vectors (i.e. Morgan substructure or N-gram amino acid sequence) into a sentence vector (i.e. compound or protein). Since the number of sum operations is dependent on the number of words in the sentence, applying these operations can significantly alter the embedded value of the sentence, regardless of the actual meaning of the sentence. To prevent information distortion, the sum operation in Mol2vec and ProtVec should be improved. Therefore, in the next section, we first refine the sum operation.

#### Improving Mol2vec and ProtVec

To refine the sum operation in Mol2vec and ProtVec, we tested two types of weighted average operations, which are arithmetic mean and Term Frequency Inverse Document Frequency (TF-IDF) [47]. The former sets the weight of each word according to the length of the sentence while the latter sets the weight of each word by TF-IDF (see "Methods and materials" section). Table 2 shows the predictive performance of nine combinations of feature pairs using the original methods and the proposed methods for both Mol2vec and ProtVec. The proposed methods performed better than original methods with the exception of the arithmetic mean method on proteins. The best method for Mol2vec is the arithmetic mean method having an average MCC of 0.659 ± 0.013 and an average ROC 0.906 ± 0.004, compared to TF-IDF weighted average method (MCC of 0.657 ± 0.013 and ROC of 0.903 ± 0.002), and the original methods (MCC of 0.649 ± 0.014 and ROC of 0.903 ± 0.006). For ProtVec, the TF-IDF weighted average method outperformed the other models with an average MCC of 0.673 ± 0.04 and an average ROC of 0.909 ± 0.003. Among all of the usage of Mol2vec and ProtVec, the best performing feature pair is arithmetic mean method for the former and TF-IDF weighted average method for the latter, where the usage of the pair showed an MCC of 0.678 ± 0.002 and a ROC of 0.912 ± 0.002.

**Table 2:** Comparison of $SC_4$’s performance obtained by different methods in Mol2vec and ProtVec

Mol2vec	ProtVec	MCC	ROC
Mean	Sum	0.652 (± 0.004)	0.905 (± 0.002)
Mean	Mean	0.648 (± 0.003)	0.902 (± 0.003)
Mean	TF-IDF	0.678 (± 0.002)	0.912 (± 0.002)
TF-IDF	Sum	0.651 (± 0.003)	0.904 (± 0.003)
TF-IDF	Mean	0.644 (± 0.002)	0.901 (± 0.002)
TF-IDF	TF-IDF	0.674 (± 0.004)	0.905 (± 0.002)
Sum	Sum	0.642 (± 0.005)	0.900 (± 0.003)
Sum	Mean	0.636 (± 0.003)	0.898 (± 0.003)
Sum	TF-IDF	0.668 (± 0.002)	0.911 (± 0.002)

It is observed that these improved methods can more accurately capture the contents of each compound and protein. The sentences (i.e. compounds and proteins) within each document (i.e. bioactivity dataset) have specific contexts and characteristics, which the entire corpus set (i.e. ZINC and UniProt) cannot represent. In particular, TF-IDF assigns a weight to each word in a sentence, so that TF-IDF weighted average method is able to more finely capture the characteristics and contexts inherent in the document.

In the case of Mol2vec, the TF-IDF weighted average method has a slightly lower performance than the arithmetic mean method. It seems that the TF-IDF weights from a specific document can be used to bias the information toward the document and reduce the generalization performance. In summary, all words were first embedded within the whole corpus, and then sentences were represented by weighting each word through a document. As a result, $SC_4$ performed better than original one, where MCC increased to 0.678 from 0.642 and ROC increased to 0.912 from 0.900.

#### Comparing the performance of multi-channel models

**Fig. 3:**
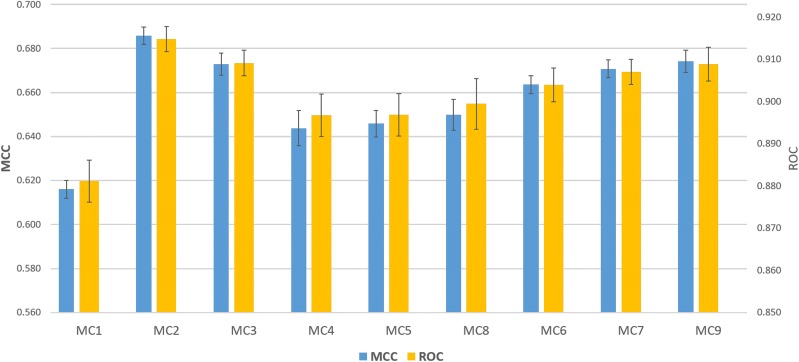
Comparison of predictive performance between *MCPINN*. On the left y-axis the MCC is shown, while on the right y-axis the ROC score is shown and error bars indicate SEM. Mean MCC is 0.658 (± 0.02) and mean ROC is 0.902 (± 0.009)

To figure out the synergy effects of a combination of both low and high level representation, the nine *MCPINN* models based on multi-channel feature pairs are evaluated as shown in Table 1. In order to improve the readability of this paper, the three multi-channel features are abbreviated as follows: ProtVec with amino acid sequences is ProtVec$_{AA}$, Mol2vec with SMILES strings is Mol2vec$_{SS}$, ECFP with SMILES strings is ECFP$_{SS}$. It is observed that the effect of multi-channel was different between proteins and compounds, as shown in Fig. 3. In the case of protein features, it was observed that the usage of ProtVec$_{AA}$ performed better than the others (average MCC of 0.658 ± 0.03 vs. 0.649 ± 0.03 and average ROC of 0.902 ± 0.02 vs. 0.897 ± 0.02). End to end learning channel with Dilated CNN seems to mainly represent the order (sentence level) of the amino acid sequence, while ProtVec channel represents the importance (word level) of each amino acid sequence in the protein. This suggests that the proposed multi-channel architecture can utilize both channels to capture features from both sentence and word perspectives for proteins.

Contrary to expectations, multi-channel models for compounds demonstrated very different results between the usage of ECFP$_{SS}$ and Mol2vec$_{SS}$. For example, the usage of ECFP$_{SS}$ performed only slightly better than the usage of ECFP (MCC of 0.670 ± 0.004 vs. 0.669 ± 0.005 and ROC of 0.907 ± 0.002 and 0.906 ± 0.003). Moreover, the models using Mol2vec$_{SS}$ performed worse than the models using Mol2vec, where the average MCC dropped to 0.65 (± 0.002) from 0.68 (± 0.006) and the average ROC dropped to 0.89 (± 0.001) from 0.91 (± 0.003). In addition, the usage of Mol2vec$_{SS}$ also resulted in lower training performance than ECFP$_{SS}$, where the average training performance was an MCC of 0.97 ± 0.006 for the models using ECFP$_{SS}$ and an MCC of 0.95 ± 0.007 for the models using Mol2vec$_{SS}$. Therefore, a careful selection of representations is required to achieve better performance.

These results suggest that the richness of the features of compounds highly depend on the base representations. For example, compounds are represented in the form of a two-dimensional graph as the raw data for ECFP and Mol2vec, where they divide the graph into substructures and define each part to extract compound features. In contrast, Dilated CNN extracts features from an one-dimensional SMILES strings and it seems to capture less generalized representations from the strings compared to the representations from ECFP and Mol2vec. In this study, sequence data was used for the multi-channel architecture but there are a variety of other data types that can be embedded and used for a new channel. Therefore, the more embedding methods applied to a variety of data types such as graphs [48], heterogeneous networks [49], and nodes [50], the more biological and molecular information (i.e. pathway and drug–drug interactions) can be fully utilized for drug discovery, poly-pharmacology, side-effect prediction, and drug resistance.

#### Ranking the features and models

**Fig. 4:**
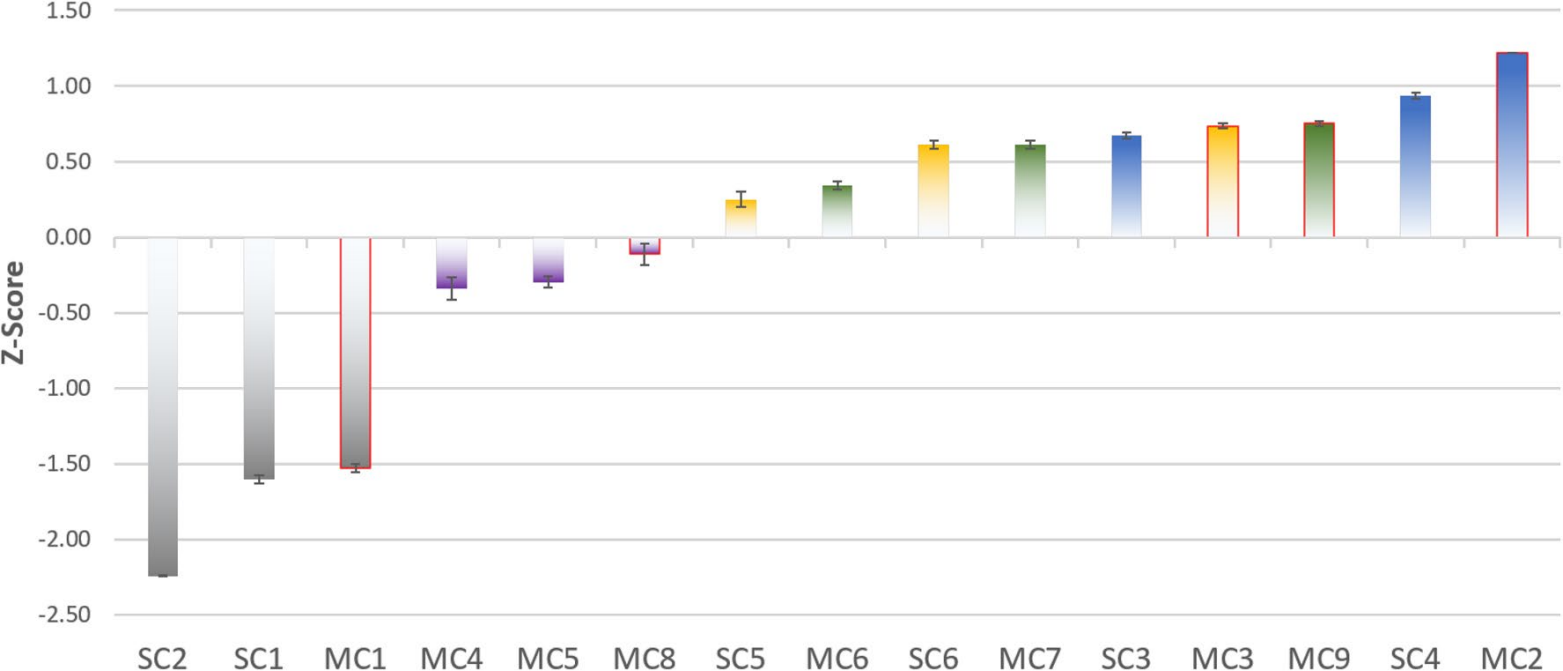
Comparison of the mean z-scores obtained by the different models and error bars indicate SEM. Bars are colored by compound features, which are blue bars for Mol2vec, green bars for ECFP$_{SS}$, yellow bars for ECFP, purple bars for Mol2vec$_{SS}$, and grey bars for SMILES. The bars highlighted with red border indicate the usage of ProtVec$_{AA}$, which demonstrates better performance than other protein features

We compared and ranked fifteen models including the six *SCPINN* models and the nine *MCPINN* models introduced above. To compare between the models, we calculated two z-scores for each model and metric (MCC and ROC) and averaged them as shown in Fig. 4 and Table 3. To verify the validity of the difference between the z-scores, the following statistical tests were performed: the paired Student’s *t* Test and the *F* Test.

**Table 3:** Comparison of performance between models expressed as z-scores per experiment

Model	MCC	ROC	Average	SEM
MC$_{2}$	1.22	1.22	1.22	0.001
SC$_{4}$	0.91	0.95	0.93	0.020
MC$_{9}$	0.77	0.73	0.75	0.017
MC$_{3}$	0.72	0.75	0.74	0.018
SC$_{3}$	0.69	0.65	0.67	0.020
MC$_{7}$	0.64	0.58	0.61	0.027
SC$_{6}$	0.64	0.58	0.61	0.030
MC$_{6}$	0.36	0.32	0.34	0.027
SC$_{5}$	0.30	0.20	0.25	0.050
MC$_{8}$	− 0.18	− 0.04	− 0.11	0.069
MC$_{5}$	− 0.34	− 0.26	− 0.30	0.038
MC$_{4}$	− 0.42	− 0.27	− 0.34	0.074
MC$_{1}$	− 1.50	− 1.55	− 1.53	0.027
SC$_{1}$	− 1.58	− 1.63	− 1.60	0.027
SC$_{2}$	− 2.24	− 2.25	− 2.24	0.004

Among the chemical features, the usage of Mol2Vec showed the best performance with an average z-score of $0.94 \pm 0.01$, compared to ECFP$_{SS}$ ($0.57 \pm 0.02$), ECFP ($0.53 \pm 0.02$), Mol2vec$_{SS}$ ($-\,0.25 \pm 0.06$), and SMILES ($-\,1.79 \pm 0.02$). For the Student’s *t* test, the usage of Mol2vec and SMILES are shown to significantly differ from all other features with a *p* value $<0.05$. Likewise, the usage of ECFP$_{SS}$ and ECFP differs significantly from all features with a *p* value $< 0.05$ with the exception of the usage of Mol2vec$_{SS}$, where the *p* value is 0.06 and 0.07 respectively (Additional file 1: Table S3). For the *F* Test, the differences in variances are also noticeable from all features with a *p* value < 0.05, with the exception of ECFP and ECFP$_{SS}$, where the *p* value is 0.38 for each other (Additional file 1: Table S4). Therefore, Mol2vec and Mol2vec$_{SS}$ showed significant differences in performance of both mean and variance, while ECFP and ECFP$_{SS}$ showed significant differences in mean performance.

Among the protein features, the usage of ProtVec$_{AA}$ outperformed the other features with an average z-scores of 0.21 ($\pm \,0.009$), compared to ProtVec ($-\,0.14 \pm 0.008$) and AA sequence ($-\,0.08 \pm 0.001$). It is observed that the usage of ProtVec$_{AA}$ performed better than the others in terms of means and variances with a *p* value < 0.05, while ProtVec and AA sequence did not differ significantly (*p* value is 0.21 and 0.06 for the means and variances respectively (Additional file 1: Tables S5, S6). It is observed that there are considerable synergy effects of multi-channel for proteins. Therefore, these statistical results indicate that the usage of Mol2vec and ProtVec$_{AA}$ outperformed the usage of the other features.

The best model was $MC_2$ with a z-score of 1.22 ($\pm \,0.001$), followed by $SC_4$ with a z-score of 0.93 ($\pm \,0.020$), and $MC_9$ with a z-score of 0.75 (± 0.017). It is observed that there were significant differences between the highest model and the lowest model compared to the other models (as can be seen in Additional file 1: Tables S7, S8). For example, for the Student’s *t* test $MC_2$ and $SC_2$ were shown to significantly differ from all other models with a *p* value < 0.05. Likewise in variance $MC_2$ and $SC_2$ were significantly different from the other models with the *p* value < 0.05. So far we have only looked into the highest performance. In the next section we look further into initial performance and the speed of convergence.

### Comparing convergence speed

In addition to the maximum performance, also noticeable are the differences in initial performance and convergence speed between *SCPINN* and *MCPINN*. Initial performance was measured by the performance at the first epoch and the speed of convergence was measured by the actual run time at $98\%$ of the highest performance of the model. In order to compare the convergence speed of each model more precisely, we mainly measured actual run time and secondarily labeled the number of epochs. For more information about convergence speed against training epochs, refer to the Additional file 1: Figure S3. The machine specifications for the experiments are described in "Methods and materials" section—Hardware used. There are more thresholds that were tested for convergence performance such as $95\%$, $98\%$, and $99\%$ in Additional file 1: Table S9. The top 3 performing models ($MC_2$, $SC_4$, and $MC_9$) and baseline model ($SC_1$) were compared. Each model showed differences in the number of parameters, training time on an epoch, and the performance but there appears to be no direct correlations between them (as can be seen Additional file 1: Figures S4 and S5).

**Fig. 5:**
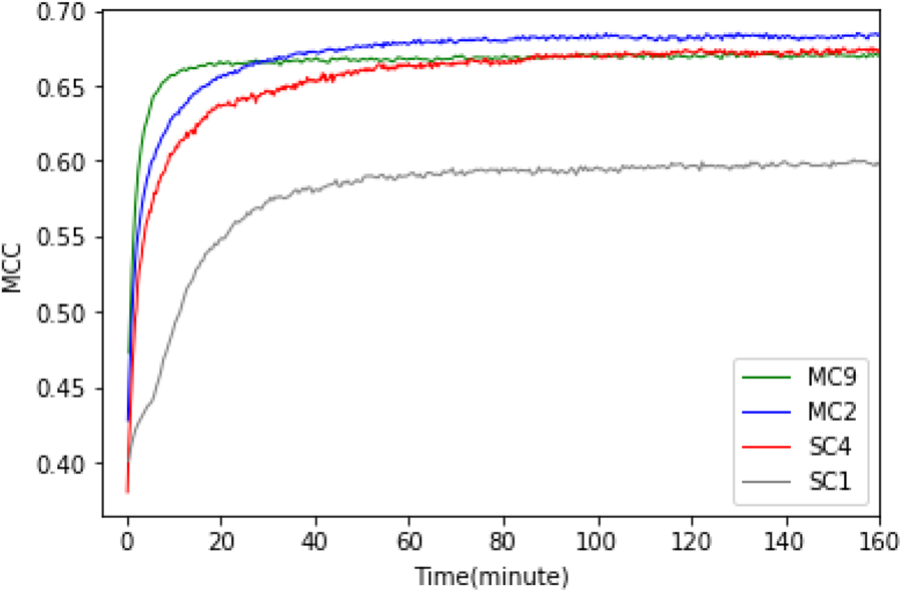
Comparison of convergence performance between two *MCPINN* and two *SCPINN*. The plot shows the Matthews Correlation Coefficient of models on y-axis against the actual training time in minutes on the x-axis

It is observed that *MCPINN* performed better than *SCPINN* in terms of initial performance and convergence speed as shown in Fig. 5. Initial performance was an MCC of 0.47 ± 0.004 for $MC_9$, 0.43 ± 0.005 for $MC_2$, 0.40 ± 0.003 for $SC_1$, and 0.38 ± 0.016 for $SC_4$. The time it took to reach $98\%$ of the highest performance was 11 min (18 epochs) for $MC_9$, 41 min (113 epochs) for $MC_2$, 50 min (102 epochs) for $SC_1$, and 55 min (201 epochs) for $SC_4$. $SC_4$ and $MC_9$ showed the most contrasting differences in the convergence speed and the highest performance. Even though the former performed a little better than the latter in performance with an MCC of 0.678 versus 0.674, it took 104 min to outperform the latter.

While the exact cause in these differences cannot be proven, it seems that low-level representations from Dilated CNNs contributed to a non-negligible portion in these differences between the models. Because it is worthwhile examining these differences between the models, let us discuss these phenomena in the perspective of the information bottleneck (IB) theory of deep learning [51]. The authors claim that “DNNs undergo two distinct phases which consist of an initial fitting/memorizing phase and a subsequent compression/forgetting phase, which is related to the high generalization performance of DNNs” [51]. In this point of view, following explanations can help account for differences in convergence speed: (1) multi-channel architecture can help to construct better representations and reduce the length of two phases because there is little information to be compressed or forgotten. (2) single-channel architecture generally need more training to discover appropriate representations for both fitting phase and compression phase, because there are not enough features. In summary, multi-channel architecture can improve convergence speed as well as the performance.

### Exploring the potential of transfer learning

While this study has focused on representation ability of *MCPINN* in terms of the performance and convergence speed, this section further explores the transferable ability of *MCPINN* to generalize representations from training tasks to related testing tasks. To test whether *MCPINN* can capture general information for compounds and proteins, we pretrained $MC_2$ on the benchmark dataset and finetuned the model on Tox21 dataset [46]. In order to improve the readability of this section, the pretrained models are abbreviated as follows: $PM_{i}$, where *i* is the number of epochs pretrained on training task, so non-pretrained model is $PM_0$. $PM_{i}$ was finetuned on the Tox21 training set with early stopping on the validation set and evaluated on the test set, where the Tox21 dataset was split as suggested by DeepChem [52].

It should be noted that the two datasets are distinct, where the benchmark dataset is based on biophysics, while the Tox21 dataset is based on physiology [53]. The benchmark dataset, obtained from ChEMBL [41], focused on bioactivity of small molecules, while Tox21 measured toxicity results in nuclear receptor and stress response pathways in human body. Because Tox21 dataset is strongly imbalanced dataset with the percentage of positives being 7.49% (5957 positives from 79,585 all data points), the performance of models was measured using MCC and Precision–Recall AUC (PRC) instead of ROC, where PRC can provide more accurate prediction when applied to imbalanced classification scenarios [54].

**Fig. 6:**
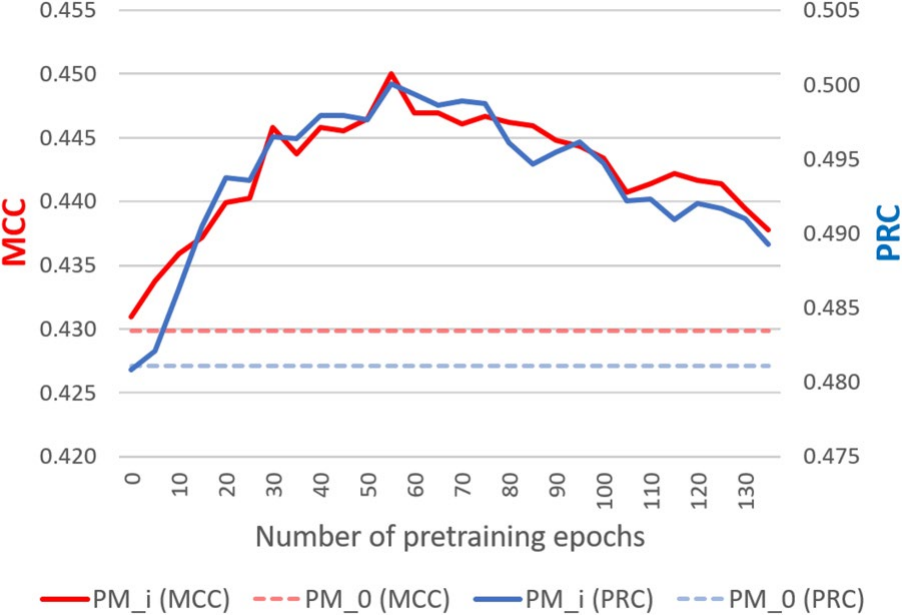
Comparison of finetuning performance between different pretrained models ($PM_i$), where *i* is the number of pretraining epochs. On the left y-axis the MCC is shown, while on the right y-axis the PRC score is shown against the number of pretraining epochs on x-axis

To investigate the potential of transferability of *MCPINN*, we have compared the performance of the models pretrained in different epochs in terms of highest performance, initial performance, and convergence speed [55, 56]. First, pretrained models performed higher than non-pretrained model. The non-pretrained model $PM_{0}$ had an MCC of 0.43 and a PRC of 0.48 as shown in Fig. 6. The pretrained models from $PM_{30}$ to $PM_{110}$ outperformed $PM_{0}$ with a paired *t* test *p* value < 0.05 for both MCC and PRC with an exception of $PM_{85}$, where the *p* value was 0.053 (Additional file 1: Table S10). It is observed that the overall performance of the models rose up to $PM_{55}$ and then declined, where it seems that the decline is because of overfitting on the training task dataset.

**Fig. 7:**
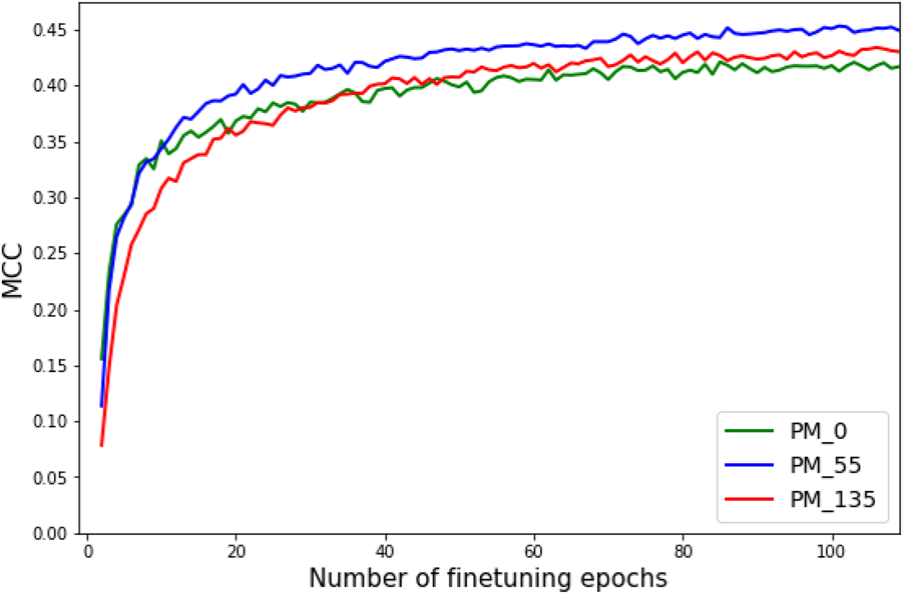
Comparison of convergence speed between models $PM_{i}$, where *i* is the number of pretraining epochs. The plot shows the MCC of models on y-axis against the number of finetuning epochs on x-axis. There were small differences in convergence speed between models

In contrast, there were small differences in initial performance and convergence speed between the models. We looked into the finetuning phase of the three models including $PM_{0}$, $PM_{55}$, and $PM_{135}$, in order to investigate the generalization performance according to the number of pretraining epochs, As shown in Table 4 and Fig. 7, $PM_{0}$ performed slightly better than other models until finetuning epoch 10, but the performance became lower than other models as finetuning continued. For example, initial performance was an MCC of 0.16 ± 0.03 for $PM_{0}$, 0.11 ± 0.02 for $PM_{55}$, and 0.08 ± 0.03 for $PM_{135}$. After finetuning epoch 11, $PM_{55}$ started to outperform $PM_{0}$ and $PM_{135}$ did so after finetuning epoch 40. In addition to initial performance, it is observed that there were similar performance in convergence speed between models. The number of finetuning epochs to reach 95% of the highest performance was 46 finetuning epochs for $PM_{55}$, 56 finetuning epochs for $PM_{135}$, and 60 finetuning epochs for $PM_{0}$.

**Table 4:** Comparison performance between different finetuning epochs for models ($PM_i$)

Models	Finetuning epoch 1	Finetuning epoch 11	Finetuning epoch 40
$PM_{55}$	0.11 ± 0.02	0.35 ± 0.01	0.43 ± 0.01
$PM_{135}$	0.08 ± 0.03	0.32 ± 0.02	0.41 ± 0.02
$PM_{0}$	0.16 ± 0.03	0.34 ± 0.01	0.40 ± 0.01

From the results we can see there is still room for improvement. The aim of transfer learning based on PCM methods is high performance with minimum finetuning. Due to the flexibility of PCM method, *MCPINN* can predict any CPI pairs, while the performance without finetuning was poor as can be seen in initial performance of each model. Since there are still a lot of small molecules with only a small amount of bioactivity data, further study of transferring general information covering CPIs space is required.

## Conclusions

In this paper we proposed a novel multi-channel PINN (*MCPINN*) based on PCM methods to fully utilize CPI data. *MCPINN* utilizes three approaches of DNNs which are a classifier, a feature extractor, and an end-to-end learner to maximize the representation learning ability. We evaluated full combinations of feature pairs to investigate the effects of each pair. We also compared *SCPINN* and *MCPINN* in terms of initial performance and the speed of convergence. In addition to improving the models within a high quality and well balanced dataset, we explored the transferable ability of *MCPINN* to generalize representations from training tasks to related testing tasks, which consist of a strongly imbalanced dataset. To the best of our knowledge, *MCPINN* is the first method to incorporate low and high level representations in a single model.

As discussed above, our results lead to a number of conclusions. For Mol2vec and ProtVec, we suggested that a weighted average operation is a better alternative to the sum operation in representing compounds and proteins. *MCPINN* using the feature pair of ProtVec$_{AA}$ and Mol2vec outperformed all other models with statistically significant differences. The usage of ProtVec$_{AA}$ performed better than others. It suggested that a multi-channel architecture can utilize both channels to capture the order and the content of amino acid sequences. The usage of Mol2vec showed statistically significant differences from the other features for compounds. In particular, the multi-channel models using Mol2vec$_{SS}$ performed worse than the single-channel models using Mol2vec separately. Depending on the combination of compound and protein features, multi-channel models did not guarantee better performance than single-channel models, so a careful selection of representations is required to achieve better performance.

The multi-channel architecture can improve initial performance and convergence speed. It seems that the architecture can help to construct better representations and reduce the length of training phase based on memorizing phase and forgetting phase in terms of IB theory of deep learning. Additionally, we explored the potential of transferability of *MCPINN* in terms of initial performance, speed of convergence, and highest performance. Pretraining on training task improved highest performance, while it did not improve convergence speed and initial performance. It seems that there is room for improvement to transfer the generalized representations from training tasks to test task.

In conclusion, *MCPINN* can improve the representations in terms of initial performance, convergence speed, and highest performance. Moreover, we expect that more biological and molecular information can be utilized as a part of multi-channel for various tasks such as drug discovery, poly-pharmacology, side-effect prediction, and drug resistance.

## Methods and materials

### Datasets

A high quality dataset [38] was employed as a benchmark dataset for the training task. Fifteen models are evaluated on the benchmark dataset. The dataset covers 0.13% of the total available bioactivity matrix space in ChEMBL, where there are 314,767 observations from 250,412,295 possible data points produced by 204,085 compounds and 1227 protein targets. Percentage of the positives in the dataset is 54.7%. We used Tox21 dataset for the test task in transfer learning. Tox21 dataset has been used in the 2014 Tox21 Data Challenge, where there are 79,585 measurements for 8014 compounds on 12 different targets. Tox21 dataset is strongly imbalanced and the percentage of positives is 7.49%.

DNNs try to minimize differences in the distribution of data between the prediction and target due to the usage of cross entropy as loss function. Therefore, training models on imbalanced data is a challenge. One of the basic solutions is to set higher weights on the positives than the negatives. In addition, it is also difficult to appropriately split the dataset into a training set, a validation set, and a test set. Therefore, we used the data splitting method and the weight value for the positive classes as suggested by Deepchem [52].

### High level representation descriptors

We used Extended-Connectivity Fingerprints with diameter of 4 (ECFP4), Mol2vec [42], and ProtVec [43] to get high level representations. ECFPs is one of the most popular representation in cheminformatics and ECFP4 have shown promising performance among various fingerprints [57]. RDkit [58] was used for ECFP4 with 1024 dimensional binary vector.

ProtVec and Mol2vec are unsupervised machine learning approaches for embedding proteins and compounds. These algorithm are inspired by a technique called Word2Vec [59] in Natural Language Processing (NLP). As a metaphor by NLP, molecules and proteins are considered as sentences. Morgan substructures and N-gram amino acid sequences are considered as “words”, and large-scale databases such as ZINC [44], ChEMBL [41] and UniProt [45] are considered as large corpus datasets.

For the protein embedding model, we train the embedding model on protein sets obtained from UniProt (release 2017_09) and ChEMBL (version 23). All duplicate sequence and same protein id with various amino sequences are removed. The number of sequences for training embedding model is 553,195. The embedding model is based on Skip-gram model and the model is trained with following hyperparameters: dimension of the representation is 300, window size is 35, and minimum count is 2. In Tox21, the target “SR-MMP” has no amino acid sequences, so the embedding value is zeros. Molecular corpus dataset, obtained from ZINC and ChEMBL (version 23), contains about 19.9 million compounds using the approach suggested in [42], and we used a pretrained embedding model the authors proposed.

Embedded sentences are composed of the group of embedded words. We build two types of weighted average methods, which are arithmetic mean and TF-IDF weighted average method, to refine the original sum method. The original sum method is as followed:

$$\begin{aligned} S^{sum} = \sum \limits _{i=1}^N w_{i} \end{aligned}$$

where $S^{sum}$ is a embedded sentence produced by the method, *N* is the number of words in the sentence, and $w_{i}$ is a *i*th embedded word in the sentence. However, the number of sum operations is dependent on *N* of each sentence, so it can alter the embedded sentence, regardless of the actual meaning of the sentence. Instead, arithmetic mean method is as followed:

$$\begin{aligned} S^{mean} = \frac{1}{N} \sum \limits _{i=1}^N w_{i} \end{aligned}$$

where $S^{mean}$ is a embedded sentence produced by the method. This method divides each word by the length of the sentence. Therefore, the same word can have different embedded value in each sentence due to the differences in length.

Moreover, TF-IDF [47] weighted average method is as followed:

$$\begin{aligned} S^{tf{-}idf} = \sum \limits _{i=1}^N t_{w} w_{i} \end{aligned}$$

where $S^{tf{-}idf}$ is a embedded sentence produced by the method and $t_w$ stands for TF-IDF weight value for a word *w*. This method sets the importance of each word by TF-IDF, so the same word has same embedded value in every sentence. To calculate weight value of TF-IDF, scikit-learn (version 0.19) is used based on compounds and proteins in benchmark dataset and Tox21.

### Low level representation descriptors

**Fig. 8:**
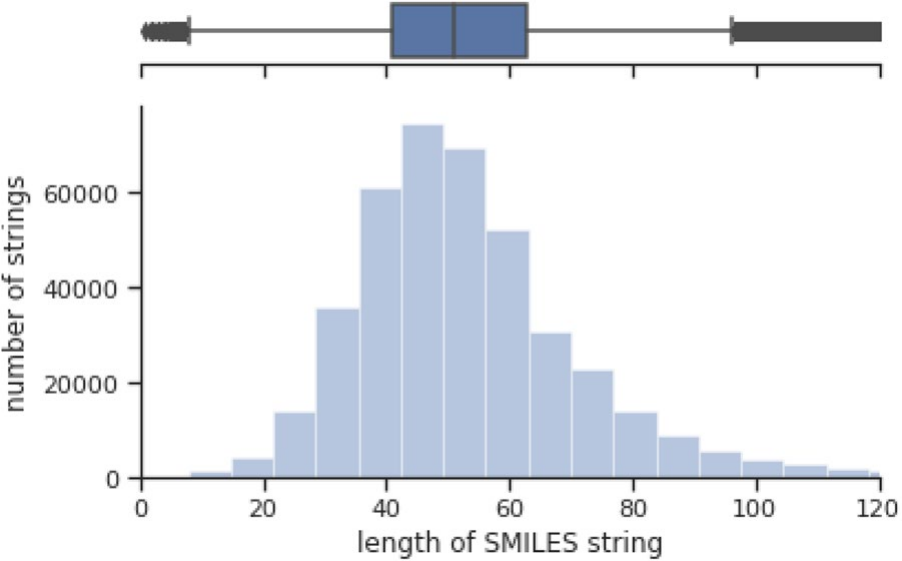
SMILES string length distribution

**Fig. 9:**
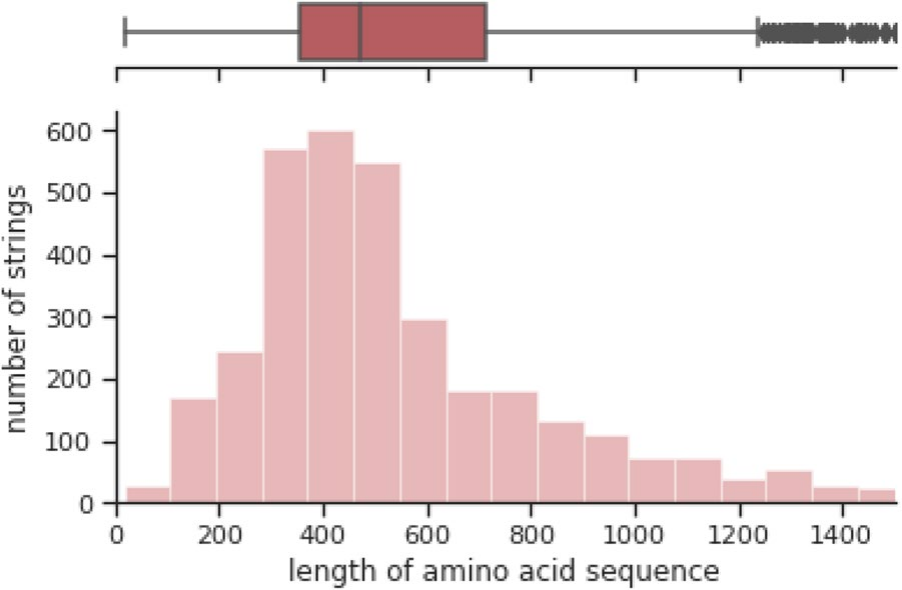
Amino acid sequence length distribution

We used low-level representation data for end-to-end learning models, where they are amino acid sequences for proteins and SMILES strings for compounds. Both sequences were tokenized and then encoded into one-hot binary vector with fixed length. Tokenizing process produced 24 single characters from the proteins and 57 single characters for SMILES as suggested in [60], where the characters are extracted from benchmark dataset and the Tox21 dataset. The tokenized strings were converted into one-hot encoded representations, which assign the corresponding single token to one and the others to zero. In order to use the sequences as an input for the machine learning model, we set the sequences to a fixed length with post truncation or zero-padding. If the sequences are longer than the fixed length, they are trimmed by removing from the end of sequences to the fixed length, unless they are filled with zero from the end of the sequences to the fixed length. In determining the fixed length of sequences, there is a trade-off between information preservation and computational efficiency. We chose the fixed length 100 for compounds and 700 for proteins, where a percentile of 75% for SMILES strings is 63.0 and a percentile of 75% for amino acid sequences is 712.2 as shown in Figs. 8 and 9.

### Transfer learning

Transfer learning focuses on whether machine learning model can transfer generalized representations from training tasks to a different but related test tasks. While there are several factors that affect finetuning methods, two important factors are generally considered [55, 56]. The factors are the size of the test task’s dataset (i.e. small or large) and similarity of test task (i.e. the content of data or classes and balance of data or classes). There are four basic strategies to finetune the pretrained models on test tasks as followed: (1) If the dataset of test task is large and the task is very similar to training task, finetuning full networks is suggested since the risk of overfitting is low. This case is expected to demonstrate promising performance. (2) If the dataset of test task is large and the task is very different from the training task, there are two options which are finetuning full networks or not pretraining the model on the training dataset. In practice, it is suggested to finetune the full networks to reduce training time. (3) If the dataset of test task is small and the task is very similar to the training task, finetuning full networks is not suggested due to the risk of overfitting. Instead, it is suggested to finetune the simple classifier to avoid overfitting. (4) If the dataset of test task is small and the task is very different from the training task, a simple classifier is not suggested due to the differences between tasks. It is suggested to initialize the top layers and freeze the other layers to finetune the layers and classifier, since the top layers contain more task-specific representations.

The benchmark dataset is four times larger than Tox21 dataset and the tasks are different in terms of biophysics versus physiology as categorized in [53]. Moreover, the benchmark dataset is balanced, while Tox21 dataset is strongly imbalanced, where the percentage of positives is 7.49% (5957 positives from 79,585 all data points). Therefore, this study corresponds to the fourth scenario. We pretrained the models on training dataset and then finetuned the pretrained models for every 5 pretraining epoch. The pretraining epoch is 140 and finetuning epoch is 200 with early stopping.

### DNNs

All DNN models are created using Keras [61] and Tensorflow [62]. We tested various architectures, parameters, and hyperparameters to optimize DNN models in initial cross-validation phase as shown in Table 5. All tested models were validated with five-fold cross-validation on validation data (20% of training data) for 400 epochs with early stopping in order to find the optimal network configuration. Then the optimal model was trained on the full training data and evaluated on test data.

**Table 5:** Architectures, parameters, and hyperparameters explored for DNNs

Base model		Value	Description
PINN	Separated layers	1, 2, 3, 4	The number of separated layers for PINN
	Concatenated layers	1, 2	The number of concatenated layers for PINN
	Number of nodes	256, 512, 1024, 2048	The number of nodes for layers
Dilated CNN	Filters	4, 8, 16, 32	The number of filters for Dilated CNN
	Kernel size	6, 8, 12, 22	The length of the convolution window for Dilated CNN
	Embedding	16, 32	Dimension of dense embedding for low level representations
LSTM, BLSTM	Units	128, 256	The units to represent hidden layers for RNN
DNN	Lr	0.0005	Initial learning rate
	Initializer	$[ - \sqrt{ 3 / fan_{in}}, \sqrt{ 3 / fan_{in}}]$	Initial weight value called Lecun uniform distribution
	Optimizer	Adam	Optimizer for stochastic gradient descent
	Weight decay	0.0, 0.00001	Learning rate decay over each update
	Activation function	ReLU, ELU	Neuron activation function
	Drop out	0.25, 0.5	The rate of drop out
	Batch	1024	Batch size for training
	Epochs_training	400	Training epochs on a training task
	Epochs_finetune	200	Finetuning epochs for a pretrained model on a test task

*Adam* is generally used in DNNs due to efficient and fast training performance because the step size is not affected by the value of the gradient. We used the hyperparameters 0.9 for $\beta _1$ and 0.999 for $\beta _2$ as suggested [63]. However, learning rate was 0.0005 and weight decay was not zero to achieve more stable training, where weight decay reduces the learning rate over each update. Since the benchmark dataset is very sparse, small batch size can mislead the training model to local optimum. Therefore, we set mini-batch size 1024 for the generalized performance as suggested [64]. All weights and biases were initialized from a uniform distribution within $[ - \sqrt{ 3 / fan_{in}}, \sqrt{ 3 / fan_{in}}]$, where $fan_{in}$ is the number of input units in the weights, which is called Lecun uniform distribution. Lecun uniform distribution performed better than random uniform distribution and truncated normal distribution in terms of performance and convergence speed, because it leads to efficient backpropagation calculations [65].

Rectified linear (ReLU) units are commonly used in DNNs because they do not suffer from vanishing gradient and their training speed is fast. However, ReLU units ignore the negative values, so there is information loss called “dying ReLU” [66]. Exponential linear units (ELU) [67] was introduced to solve the problem. ELU and ReLU are in identity function form for non-negative inputs, but for negative inputs, they are different, where if $x <0$, $f(x)= \alpha (e^x-1)$ for ELU and $f(x)=0$ for ReLU. ELU can capture information in the negative value. Therefore, we used following parameters for the final DNN models: (1) as an optimizer *Adam* with 0.9 beta 1 and 0.999 beta 2 is used as suggested [63], (2) learning rate is 0.0005, (3) number of epochs is 500, (4) mini-batch size is 1024, (5) Lecun uniform distribution, (6) the weight decay is 0.00001, (7) activation function is ELU.

#### DNNs: end-to-end learning

We built three types of end-to-end DNNs based on convolution neural networks (CNN) and recurrent neural networks (RNN). RNN is designed to learn sequential data and CNN has multiple filters which are incorporated with each other to discover various representations. These model have shown promising performance for sequential data in various domains. Among RNN models, long short-term memory (LSTM) [68] and bidirectional LSTM (BLSTM) [69] have outperformed conventional models (i.e. Hidden Markov model) and recent proposed models (i.e. Gated Recurrent Unit) over two decades [70]. LSTM is a recurrent neural network model with explicit memory cell. Due to the memory cell, LSTM can remember or forget long-term dependencies needed for tasks. The memory cell is carefully regulated by four modules, which are input gate, forget gate, output gate, and cell update. Bidirectional LSTM (BLSTM) is a variant version of LSTM. BLSTM has two LSTMs which go in opposite directions, forward and backward. The two features complement each other and contribute to performance improvement.

We used dilated convolution neural networks (Dilated CNN) [71] among end-to-end learners. Dilated CNN is a convolution neural networks with skip layers. Conventional CNN learns long-term dependency by reducing the size of the data, but it results in information loss. In contrast, Dilated CNN can learn long-term dependency efficiently with skip layers. The layers have wider receptive fields compared to conventional layers. The size of kernel is the length of the convolution window and it affects the long-term dependency of given sequences. The basic suggestion is a small kernel size (i.e. 3) to achieve efficient training and less number of parameters [72]. However, we chose larger size of kernel, since ProtVec and Mol2vec already captured the features in terms of local perspective.

The number of filters determines depth of the output volume called feature map, which is the result of the convolution layer. If the number of filters is too large the model can suffer from overfitting, otherwise the model can suffer from underfitting. In computer vision domain, the number of filter is large but we tested smaller numbers of filter due to the sparseness of CPI data space. The embedding layer is the first layer for one-hot encoded vectors. The dimension size 32 and 16 was tested, but there were little differences in performance. Therefore, the final value of network architecture and hyperparameters for Dilated CNNs were (1) the number of filters is 16, (2) the kernel size is 12, (3) an embedding layer with 16 dimension is used to reduce the number of parameters, and (4) valid padding to reduce the shape of the feature maps in each layers.

For LSTM and BLSTM, the final value of network architecture and hyperparameters were: (1) units are 256, which is the dimensionality of output, (2) set forget bias as suggested [73].

#### DNNs: pairwise input neural networks

Pairwise input neural network (PINN) is used for *MCPINN* and *SCPINN*. PINN is a variation of feedforward neural networks and is a more suitable architecture for PCM methods. It consists of separated layers with two input and concatenated layers. For *MCPINN*, all channels are merged in the concatenated layer as shown in Fig. 1. Since the separated layers are independently composed without connection from other layers, each input channel layers build representations independently for each input. Moreover, the architecture can balance the ratio of each feature by controlling the number of nodes in the last separated layers. For example, although the input dimension of ECFP is 1024 and the input dimension of ProtVec is 300, the dimension of each representation is the number of nodes in the last separated layers. In contrast, DNNs can be biased to the feature of larger input dimensions.

We normalized the high-level representations with zero mean and unit variance to achieve stable training, because outliers can degrade the performance of machine learning algorithms in terms of prediction, learning speed, and the convergence of the algorithms. In particular, many gradient based algorithms (i.e. deep learning) are often designed with the assumption that input data is nearly standardized data, which is generally obtained by subtracting the mean and scaling the data to unit variance. We tested separated layers from 1 to 4 and concatenated layer from 1 to 2. The number of concatenated layer is 1 for both *SCPINN* and *MCPINN*, but the number of each separated layers is 2 for *SCPINN* and 1 for *MCPINN* to reduce overfitting, where the parameters of end-to-end channel was added in *MCPINN*. To prevent overfitting, we used 10% dropout on initial layer and 50% on hidden layers and early stopping.

In our experiments, the final value of network architecture and hyperparameters for PINN were: (1) the number of each separated layers is 2 for *SCPINN* and 1 for *MCPINN*, (2) the number of each concatenated layer is 1, (3) the number of units in each separated layer is 1024 and 256, (4) the number of units in each concatenated layer is 256, (5) dropout rate is 0.5 (6) each features are normalized with zero mean and unit variance.

### Performance metrics

For the performance evaluation, we used three metrics, which are Matthew Correlation Coefficient (MCC), Receiver Operating Characteristic Area Under the Curve (ROC), and Precision–Recall Area Under the Curve (PRC). Above metrics are commonly used in binary classification to evaluate the quality of the performance. ROC space is determined by the false positive rate (FPR) versus true positive rate (TPR) called recall as x and y axis, where FPR and TPR is calculated by following formula: $TPR = TP / (TP+FN)$ and $FPR = FP/(FP+TN)$, where *TP* is the number of true positives, *FN* the number of false negatives, *FP* the number of false positives, and *TN* the number of true negatives. It means ROC shows relative trade-offs between true positive and false positive. The value of ROC is between 0 and + 1, where + 1 indicated perfect prediction, 0.5 means random prediction, and 0 indicates totally wrong prediction.

PRC can provide more accurate prediction when applied to imbalanced classification scenario than ROC, because PRC put more importance on the TPR in case of imbalanced dataset. ROC and PRC share TPR (recall) on same axis, but PRC uses precision for the other axis, where precision is calculated by following formula: $precision = TP / (FP+TP)$. MCC is generally regarded as being one of the best metrics because MCC is more useful than other metrics when the two classes are very different. MCC is calculated by following formula:

$$\begin{aligned} \frac{TP \times TN - FP \times FN }{ \sqrt{ (TP+FP)(TP+FN)(TN+FP)(TN+FN)}} \end{aligned}$$

The value of MCC is between $-\,1$ and + 1, where + 1 indicates perfect prediction, 0 means random prediction, and − 1 represents totally wrong prediction.

### Software used

Python (version 2.7) was used with the following libraries: Keras (version 2.2.0) and Tensorflow (1.9.0) for the neural networks, RDKit (version 2017.03.3) for the calculation of the fingerprints and descriptors, scikit-learn (version 0.19) for splitting validation, normalization and performance evaluation, SciPy (version 1.2.0) for statistical analysis including students *t* test and Fisher F test, ProtVec for the protein descriptors, and Mol2vec for the molecule descriptors.

### Hardware used

A Linux server running Ubuntu 16.04 was established for experiments. The server was equipped with a Xeon E5-2620 v4 processor, 96 GB RAM, and four NVIDIA GeForce GTX 1080 Ti for Graphics Processing Units.

## Additional file

**Additional file 1.** Supplementary results in the form of 5 Figures and 10 Tables.

## Data Availability

The source code and data supporting the conclusions of this article are available in the following link: https://github.com/mhlee0903/multi_channels_PINN.git.

## Abbreviations

AUC: area under the curve
CPI: compound–protein interaction
DNN: deep neural network
ECFP: extended-connectivity fingerprints
IB: information bottleneck
MCC: Matthews correlation coeffcient
MCPINN: multi-channel pairwise input neural networks
PCM: proteochemometrics
PINN: pairwise input neural networks
PRC: precision–recall curve
QSAR: quantitative structure–activity relationship
ROC: receiver operator characteristic
SCPINN: single-channel pairwise input neural networks
SMILES: simplifed molecular input line entry system
TF-IDF: term frequency inverse document frequency

## References

[1] Rifaioglu AS, Atas H, Martin MJ, Cetin-Atalay R, Atalay V, Doğan T (2018). Recent applications of deep learning and machine intelligence on in silico drug discovery: methods, tools and databases. Brief Bioinform.

[2] Chen R, Liu X, Jin S, Lin J, Liu J (2018). Machine learning for drug–target interaction prediction. Molecules.

[3] Ding H, Takigawa I, Mamitsuka H, Zhu S (2013). Similarity-based machine learning methods for predicting drug–target interactions: a brief review. Brief Bioinform.

[4] Kim S, Thiessen PA, Bolton EE, Chen J, Fu G, Gindulyte A, Han L, He J, He S, Shoemaker BA (2015). Pubchem substance and compound databases. Nucleic Acids Res.

[5] Walters WP (2018). Virtual chemical libraries: miniperspective. J Med Chem.

[6] Ruddigkeit L, Van Deursen R, Blum LC, Reymond J-L (2012). Enumeration of 166 billion organic small molecules in the chemical universe database GDB-17. J Chem Inf Model.

[7] Consortium U (2014). Uniprot: a hub for protein information. Nucleic Acids Res.

[8] Cao D-S, Liu S, Xu Q-S, Lu H-M, Huang J-H, Hu Q-N, Liang Y-Z (2012). Large-scale prediction of drug–target interactions using protein sequences and drug topological structures. Anal Chim Acta.

[9] Gönen M (2012). Predicting drug–target interactions from chemical and genomic kernels using bayesian matrix factorization. Bioinformatics.

[10] Scior T, Bender A, Tresadern G, Medina-Franco JL, Martínez-Mayorga K, Langer T, Cuanalo-Contreras K, Agrafiotis DK (2012). Recognizing pitfalls in virtual screening: a critical review. J Chem Inf Model.

[11] Reymond J-L, Van Deursen R, Blum LC, Ruddigkeit L (2010). Chemical space as a source for new drugs. MedChemComm.

[12] Li H, Gao Z, Kang L, Zhang H, Yang K, Yu K, Luo X, Zhu W, Chen K, Shen J (2006). Tarfisdock: a web server for identifying drug targets with docking approach. Nucleic Acids Res.

[13] Xie L, Evangelidis T, Xie L, Bourne PE (2011). Drug discovery using chemical systems biology: weak inhibition of multiple kinases may contribute to the anti-cancer effect of nelfinavir. PLoS Comput Biol.

[14] Yang L, Wang K, Chen J, Jegga AG, Luo H, Shi L, Wan C, Guo X, Qin S, He G (2011). Exploring off-targets and off-systems for adverse drug reactions via chemical-protein interactome—clozapine-induced agranulocytosis as a case study. PLoS Comput Biol.

[15] Keiser MJ, Roth BL, Armbruster BN, Ernsberger P, Irwin JJ, Shoichet BK (2007). Relating protein pharmacology by ligand chemistry. Nat Biotechnol.

[16] Campillos M, Kuhn M, Gavin A-C, Jensen LJ, Bork P (2008). Drug target identification using side-effect similarity. Science.

[17] Koutsoukas A, Simms B, Kirchmair J, Bond PJ, Whitmore AV, Zimmer S, Young MP, Jenkins JL, Glick M, Glen RC (2011). From in silico target prediction to multi-target drug design: current databases, methods and applications. J Proteom.

[18] van Westen GJ, Wegner JK, IJzerman AP, van Vlijmen HW, Bender A (2011). Proteochemometric modeling as a tool to design selective compounds and for extrapolating to novel targets. MedChemComm.

[19] Westen G (2013). Benchmarking of protein descriptor sets in proteochemometric modeling (part 1): comparative study of 13 amino acid descriptor sets. J Cheminform.

[20] Cortés-Ciriano I, Ain QU, Subramanian V, Lenselink EB, Méndez-Lucio O, IJzerman AP, Wohlfahrt G, Prusis P, Malliavin TE, van Westen GJ (2015). Polypharmacology modelling using proteochemometrics (PCM): recent methodological developments, applications to target families, and future prospects. MedChemComm.

[21] Qiu T, Qiu J, Feng J, Wu D, Yang Y, Tang K, Cao Z, Zhu R (2016). The recent progress in proteochemometric modelling: focusing on target descriptors, cross-term descriptors and application scope. Brief Bioinform.

[22] Morris GM, Goodsell DS, Halliday RS, Huey R, Hart WE, Belew RK, Olson AJ (1998). Automated docking using a Lamarckian genetic algorithm and an empirical binding free energy function. J Comput Chem.

[23] Friesner RA, Banks JL, Murphy RB, Halgren TA, Klicic JJ, Mainz DT, Repasky MP, Knoll EH, Shelley M, Perry JK (2004). Glide: a new approach for rapid, accurate docking and scoring. 1. Method and assessment of docking accuracy. J Med Chem.

[24] McGann M (2011). Fred pose prediction and virtual screening accuracy. J Chem Inf Model.

[25] Wallach I, Dzamba M, Heifets A (2015) Atomnet: a deep convolutional neural network for bioactivity prediction in structure-based drug discovery. arXiv preprint arXiv:1510.02855

[26] Bender A, Glen RC (2004). Molecular similarity: a key technique in molecular informatics. Org Biomol Chem.

[27] Nigsch F, Bender A, Jenkins JL, Mitchell JB (2008). Ligand-target prediction using winnow and naive Bayesian algorithms and the implications of overall performance statistics. J Chem Inf Model.

[28] Lowe R, Mussa HY, Nigsch F, Glen RC, Mitchell JB (2012). Predicting the mechanism of phospholipidosis. J Cheminform.

[29] Svetnik V, Liaw A, Tong C, Culberson JC, Sheridan RP, Feuston BP (2003). Random forest: a classification and regression tool for compound classification and QSAR modeling. J Chem Inf Comput Sci.

[30] Lowe R, Mussa HY, Mitchell JB, Glen RC (2011). Classifying molecules using a sparse probabilistic kernel binary classifier. J Chem Inf Model.

[31] Ma J, Sheridan RP, Liaw A, Dahl GE, Svetnik V (2015). Deep neural nets as a method for quantitative structure–activity relationships. J Chem Inf Model.

[32] Dahl GE, Jaitly N, Salakhutdinov R (2014) Multi-task neural networks for QSAR predictions. arXiv preprint arXiv:1406.1231

[33] Ramsundar B, Liu B, Wu Z, Verras A, Tudor M, Sheridan RP, Pande V (2017). Is multitask deep learning practical for pharma?. J Chem Inf Model.

[34] Iwata H, Sawada R, Mizutani S, Kotera M, Yamanishi Y (2015). Large-scale prediction of beneficial drug combinations using drug efficacy and target profiles. J Chem Inf Model.

[35] Li Z, Han P, You Z-H, Li X, Zhang Y, Yu H, Nie R, Chen X (2017). In silico prediction of drug–target interaction networks based on drug chemical structure and protein sequences. Sci Rep.

[36] Yabuuchi H, Niijima S, Takematsu H, Ida T, Hirokawa T, Hara T, Ogawa T, Minowa Y, Tsujimoto G, Okuno Y (2011). Analysis of multiple compound–protein interactions reveals novel bioactive molecules. Mol Syst Biol.

[37] Lapinsh M, Prusis P, Lundstedt T, Wikberg JE (2002). Proteochemometrics modeling of the interaction of amine g-protein coupled receptors with a diverse set of ligands. Mol Pharmacol.

[38] Lenselink EB, Ten Dijke N, Bongers B, Papadatos G, van Vlijmen HW, Kowalczyk W, IJzerman AP, van Westen GJ (2017). Beyond the hype: deep neural networks outperform established methods using a ChEMBL bioactivity benchmark set. J Cheminform.

[39] Koutsoukas A, Monaghan KJ, Li X, Huan J (2017). Deep-learning: investigating deep neural networks hyper-parameters and comparison of performance to shallow methods for modeling bioactivity data. J Cheminform.

[40] Wang C, Liu J, Luo F, Tan Y, Deng Z, Hu Q-N (2014) Pairwise input neural network for target–ligand interaction prediction. In: 2014 IEEE international conference on bioinformatics and biomedicine (BIBM). IEEE, pp 67–70

[41] Papadatos G, Gaulton A, Hersey A, Overington JP (2015). Activity, assay and target data curation and quality in the ChEMBL database. J Comput Aided Mol Des.

[42] Jaeger S, Fulle S, Turk S (2018). Mol2vec: unsupervised machine learning approach with chemical intuition. J Chem Inf Model.

[43] Asgari E, Mofrad MR (2015). Continuous distributed representation of biological sequences for deep proteomics and genomics. PLoS ONE.

[44] Irwin JJ, Sterling T, Mysinger MM, Bolstad ES, Coleman RG (2012). Zinc: a free tool to discover chemistry for biology. J Chem Inf Model.

[45] Consortium U (2018). Uniprot: the universal protein knowledgebase. Nucleic Acids Res.

[46] Program NT (2014) Tox21 challenge. https://tripod.nih.gov/tox21/challenge/. Accessed 3 Dec 2018

[47] Sparck Jones K (1972). A statistical interpretation of term specificity and its application in retrieval. J Doc.

[48] Ribeiro LF, Saverese PH, Figueiredo DR (2017) Struc2vec: learning node representations from structural identity. In: Proceedings of the 23rd ACM SIGKDD international conference on knowledge discovery and data mining. ACM, pp 385–394

[49] Dong Y, Chawla NV, Swami A (2017) Metapath2vec: scalable representation learning for heterogeneous networks. In: Proceedings of the 23rd ACM SIGKDD international conference on knowledge discovery and data mining. ACM, pp 135–144

[50] Grover A, Leskovec J (2016) Node2vec: scalable feature learning for networks. In: Proceedings of the 22nd ACM SIGKDD international conference on knowledge discovery and data mining. ACM, pp 855–864 10.1145/2939672.2939754 PMC5108654 27853626

[51] Saxe AM, Bansal Y, Dapello J, Advani M, Kolchinsky A,Tracey BD, Cox DD(2018) On the information bottleneck theory of deeplearning. In: 6th International Conference on Learning Representations, ICLR 2018, Vancouver, BC, Canada, April 30 - May 3, 2018, Conference Track Proceedings. https://openreview.net/forum?id=ry_WPG-A-

[52] Ramsundar B, Eastman P, Leswing K, Walters P, Pande V (2019). Deep learning for the life sciences.

[53] Wu Z, Ramsundar B, Feinberg EN, Gomes J, Geniesse C, Pappu AS, Leswing K, Pande V (2018). Moleculenet: a benchmark for molecular machine learning. Chem Sci.

[54] Saito T, Rehmsmeier M (2015). The precision–recall plot is more informative than the ROC plot when evaluating binary classifiers on imbalanced datasets. PLoS ONE.

[55] Olivas ES (2009). Handbook of research on machine learning applications and trends.

[56] Goodfellow I, Bengio Y, Courville A, Bengio Y (2016). Deep learning.

[57] Bender A, Jenkins JL, Scheiber J, Sukuru SCK, Glick M, Davies JW (2009). How similar are similarity searching methods? A principal component analysis of molecular descriptor space. J Chem Inf Model.

[58] RDKit: open-source cheminformatics. http://www.rdkit.org. Accessed 11 Apr 2018

[59] Mikolov T, Sutskever I, Chen K, Corrado GS, Dean J (2013) Distributed representations of words and phrases and their compositionality. In: Advances in neural information processing systems, pp 3111–3119

[60] Gómez-Bombarelli R, Wei JN, Duvenaud D, Hernández-Lobato JM, Sánchez-Lengeling B, Sheberla D, Aguilera-Iparraguirre J, Hirzel TD, Adams RP, Aspuru-Guzik A (2018). Automatic chemical design using a data-driven continuous representation of molecules. ACS Cent Sci.

[61] Chollet F et al (2015) Keras. https://keras.io/. Accessed 27 July 2018

[62] Abadi M, Agarwal A, Barham P, Brevdo E, Chen Z, Citro C, Corrado GS, Davis A, Dean J, Devin M, Ghemawat S, Goodfellow I, Harp A, Irving G, Isard M, Jia Y, Jozefowicz R, Kaiser L, Kudlur M, Levenberg J, Mané D, Monga R, Moore S, Murray D, Olah C, Schuster M, Shlens J, Steiner B, Sutskever I, Talwar K, Tucker P, Vanhoucke V, Vasudevan V, Viégas F, Vinyals O, Warden P, Wattenberg M, Wicke M, Yu Y, Zheng X (2015) TensorFlow: large-scale machine learning on heterogeneous systems. Software available from tensorflow.org. https://www.tensorflow.org/. Accessed 27 July 2018

[63] Kingma DP, Ba J (2014) Adam: a method for stochastic optimization. arXiv preprint arXiv:1412.6980

[64] Keskar NS, Mudigere D, Nocedal J, Smelyanskiy M, Tang PTP (2016) On large-batch training for deep learning: generalization gap and sharp minima. arXiv preprint arXiv:1609.04836

[65] LeCun Yann A., Bottou Léon, Orr Genevieve B., Müller Klaus-Robert (2012). Efficient BackProp. Lecture Notes in Computer Science.

[66] Xu B, Wang N, Chen T, Li M (2015) Empirical evaluation of rectified activations in convolutional network. arXiv preprint arXiv:1505.00853

[67] Clevert D-A, Unterthiner T, Hochreiter S (2015) Fast and accurate deep network learning by exponential linear units (elus). arXiv preprint arXiv:1511.07289

[68] Hochreiter S, Schmidhuber J (1997). Long short-term memory. Neural Comput.

[69] Graves A, Schmidhuber J (2005). Framewise phoneme classification with bidirectional LSTM and other neural network architectures. Neural Netw.

[70] Greff K, Srivastava RK, Koutník J, Steunebrink BR, Schmidhuber J (2017). LSTM: a search space odyssey. IEEE Trans Neural Netw Learn Syst.

[71] Van Den Oord A, Dieleman S, Zen H, Simonyan K, Vinyals O, Graves A, Kalchbrenner N, Senior AW, Kavukcuoglu K (2016) Wavenet: a generative model for raw audio. In: The 9th ISCA Speech Synthesis Workshop, Sunnyvale, CA, USA, 13–15 September 2016, p.125

[72] Szegedy C, Vanhoucke V, Ioffe S, Shlens J, Wojna Z (2016) Rethinking the inception architecture for computer vision. In: Proceedings of the IEEE conference on computer vision and pattern recognition, pp 2818–2826

[73] Jozefowicz R, Zaremba W, Sutskever I (2015) An empirical exploration of recurrent network architectures. In: International conference on machine learning, pp 2342–2350

