# Supplementary material for: Multi-channel PINN: investigating scalable and transferable neural networks for drug discovery
Source: J Cheminform. 2019 Jul 9;11:46. doi: 10.1186/s13321-019-0368-1 (PMC6617572; doi:10.1186/s13321-019-0368-1)
Supplement: Supplementary file 1 — Additional file 1. Supplementary results in the form of 5 Figures and 10 Tables. [file 13321_2019_368_MOESM1_ESM.docx]

**Multi-channel PINN: Investigating Scalable and Transferable Neural Networks for Drug Discovery**

Munhwan Lee^1^, Hyeyeon Kim^1^, Hyunwhan Joe^1^ and Hong-Gee Kim^1^

^1­^Biomedical Knowledge Engineering Lab., Seoul National University, Seoul, Republic of Korea.

Author email addresses: Mh Lee [munhwanlee@snu.ac.kr](mailto:munhwanlee@snu.ac.kr) ,
Hy Kim [ann4645@snu.ac.kr](mailto:ann4645@snu.ac.kr) , H Joe [hyunwhanjoe@snu.ac.kr](mailto:hyunwhanjoe@snu.ac.kr) and
Hg Kim [hgkim@snu.ac.kr](mailto:hgkim@snu.ac.kr)

Figure S1: Plot of performance of end to end models page 3

Figure S2: Convergence speed of end to end models page 4

Figure S3. Convergence speed of top3 models and the baseline model in terms of an epoch page 5

Figure S4. Actual run time plotted against the number of parameters page 6

Figure S5. Performance plotted against the number of parameters and training time page 7

Table S1: Comparison of the number of parameters and performance between FFNN and PINN page 8

Table S2: Results for the models using concatenating both ECFP and Mol2vec and baseline page 9

Table S3 : Overview of *p* values obtained with Students *T* Test on compound features page 10

Table S4 : Overview of p values obtained with Fisher F test on compound features page 11

Table S5 : Overview of *p* values obtained with Students *T* Test on protein features page 12

Table S6 : Overview of p values obtained with Fisher F test on protein features page 13

Table S7 : Overview of *p* values obtained with Students *T* Test on all models page 14

Table S8 : Overview of p values obtained with Fisher F test on all models page 15

Table S9: Overview of convergence speed for different thresholds page 16

Table S10: Overview of *p* values obtained with a paired *t* test on finetuned models page 17


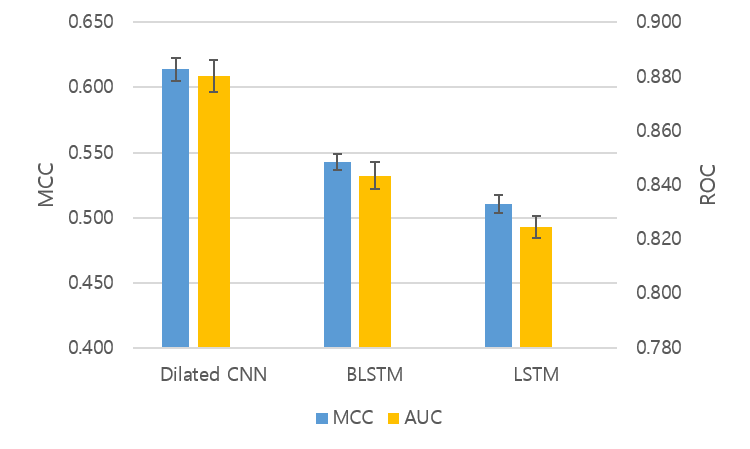


Figure S1: Performance of end to end models including Dilated CNN, BLSTM, and LSTM. The models used amino acid sequences for proteins and SMILES strings for compounds. The plot shows the Mathew Correlation Coefficient (MCC) and Receiver Operating Characteristic Area Under the Curve (AUC) for each model. Dilated CNN outperformed the others. These results suggest that multiple filters can help improve performance for sequential data from low level representations, and in addition, long term dependency can. All models can represent the long term dependency, but Dilated CNN is the only model with the above two abilities. Dilated CNN has multiple filters (i.e. 16) called kernels, which produce various representations from the same input. BLSTM has a single filter for each forward and backward direction and LSTM has only a single filter for the forward and backward direction.


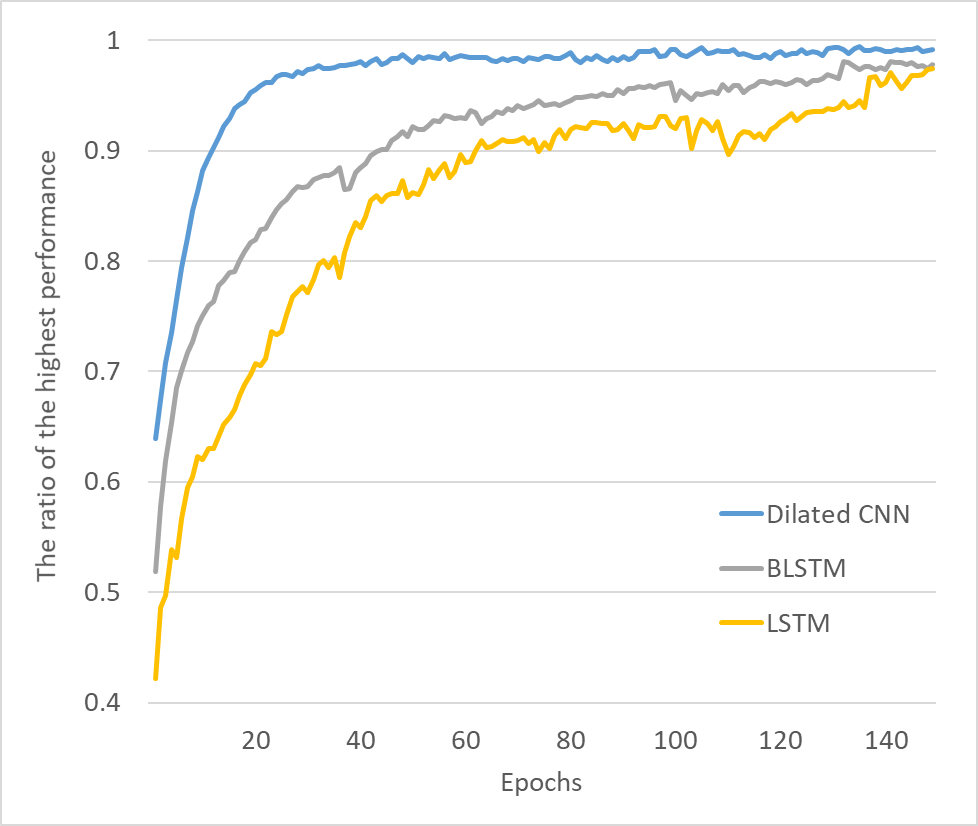


Figure S2: Convergence speed of end to end models including Dilated CNN, BLSTM, and LSTM. The models used amino acid sequences for protein and SMILES strings for compound. The plot shows ratio of the highest performance along training phase. Dilated CNNs started with better initial performance and performed with faster training speed than the other models during almost training steps. In addition to the initial performance, it is observed that Dilated CNNs outperformed other models in terms of faster convergence ratio. For example, Dilated CNNs reached 98% percent of the highest performance at 39 epochs and remained stable. In contrast, BLSTM touched it few times after 125 epochs and LSTM touched it after 173 epochs.


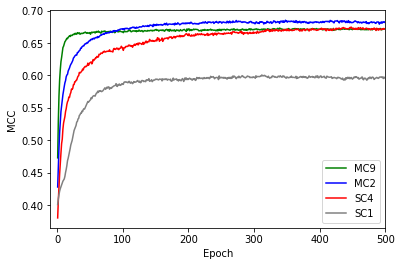


Figure S3. Convergence speed of top3 models and the baseline model in terms of an epoch. The performance is measured with the Matthews Correlation Coefficient. MC9 outperformed all other models in convergence speed, where MC9 used the feature pair of {SMILES+ECFP, AA_SEQ+ProtVec}.


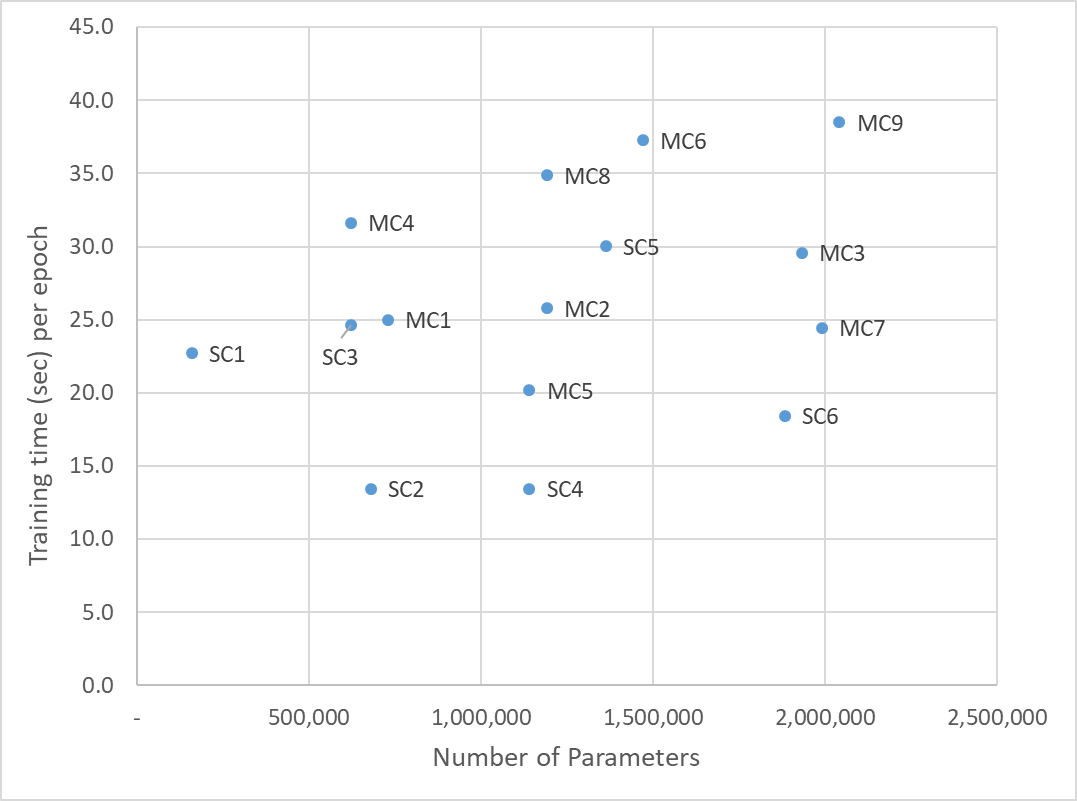


Figure S4. Training time per epoch plotted against the number of parameters. The actual run time is measured by the machine specs in Method section “Hardware used”. There appears to be no direct correlation between actual run time and the number of parameters (R^2^ 0.02).


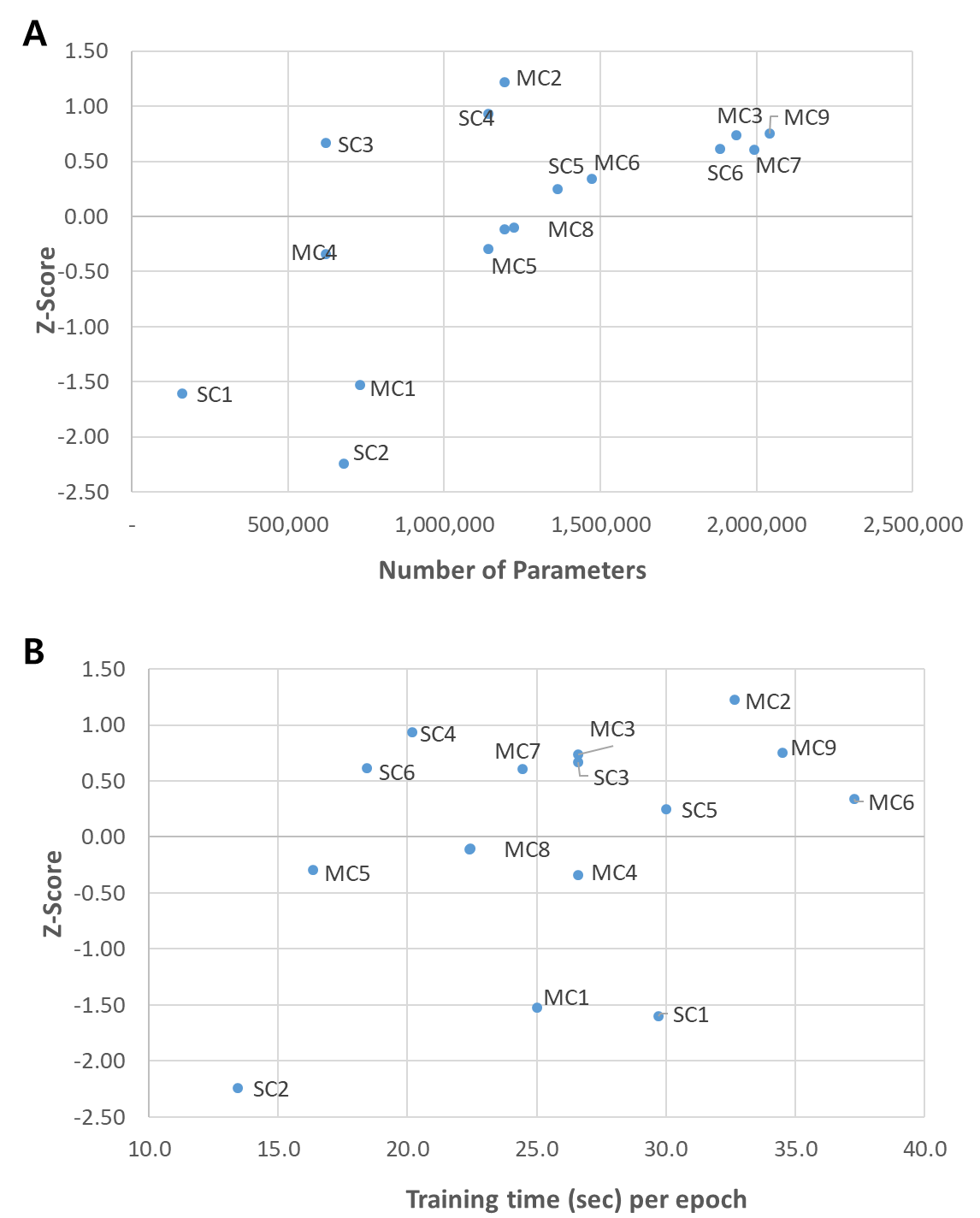


Figure S5. Performance as z-score plotted against (A) the number of parameters and (B) training time per epoch. Above number of parameters, training time per epoch, z-score is based on the final model. The actual run time is measured by the machine specs in Method section “Hardware used”. There appears to be no direct correlation between z-score and the number of parameters (R^2^ 0.43). Also, there appears to be no direct correlation between z-score and actual training time (R^2^ 0.14).

Table S1 : Comparison the number of parameters and performance between FFNN and PINN

|  | Separated  layers | Fully  connected  layers | Number of  parameters | MCC | ROC |
| --- | --- | --- | --- | --- | --- |
| PINN | 2 | 1 | 1,273,090 | 0.678 | 0.912 |
| FFNN |  | 3 | 2,411,778 | 0.676 | 0.911 |

Comparison between PINN model and FFNN model. Each model uses Mol2vec and ProtVec. There is no difference in performance between models. However, the number of parameters in PINN is 52.5% of FFNN.

Table S2: Results for the models using compound feature of concatenating ECFP and Mol2vec

| **Baseline compound feature** | **Compound feature** | **Protein feature** | **MCC** | **Mean of MCC diff. (concat-baseline)** | **ROC** | **Mean of ROC diff (concat-baseline)** |
| --- | --- | --- | --- | --- | --- | --- |
| Mol2vec | Concat | AA | 0.666 | 0.000 | 0.906 | 0.001 |
|  |  | ProtVec | 0.668 | -0.006 | 0.901 | -0.004 |
|  |  | AA+ProtVec | 0.674 | -0.012 | 0.909 | -0.006 |
| SMILES +ECFP | SMILES +Concat | AA | 0.668 | 0.004 | 0.905 | 0.001 |
|  |  | ProtVec | 0.672 | 0.001 | 0.907 | 0.000 |
|  |  | AA+ProtVec | 0.674 | 0.000 | 0.910 | 0.001 |

The performance of models using concatenated feature of Mol2vec and ECFP (concat model) and baseline models using Mol2vec and ECFP separately. There is no significantly differences between concat models and baseline models.

Table S3: Overview of *p* values obtained with Students T test on compound features

|  | SMILES | SMILES+ Mol2vec | ECFP | SMILES+ ECFP | Mol2vec |
| --- | --- | --- | --- | --- | --- |
| SMILES | 1.00 | 0.03 | 0.00 | 0.00 | 0.00 |
| SMILES+ Mol2vec |  | 1.00 | 0.07 | 0.06 | 0.03 |
| ECFP |  |  | 1.00 | 0.01 | 0.03 |
| SMILES+ ECFP |  |  |  | 1.00 | 0.04 |
| Mol2vec |  |  |  |  | 1.00 |

Above p values are based on the MCC and AUC. Color thresholds are 0.05 (blue), 0.07 (white), and 0.3 (red). The values between the thresholds are expressed in the middle color such as light blue for 0.06, which is between blue and white.

Table S4: Overview of *p* values obtained with Fisher F test on compound features

| compound | SMILES | SMILES+ Mol2vec | ECFP | SMILES+ ECFP | Mol2vec |
| --- | --- | --- | --- | --- | --- |
| SMILES | 1.00 | 0.00 | 0.00 | 0.00 | 0.00 |
| SMILES+ Mol2vec |  | 1.00 | 0.01 | 0.01 | 0.00 |
| ECFP |  |  | 1.00 | 0.36 | 0.00 |
| SMILES+ ECFP |  |  |  | 1.00 | 0.00 |
| Mol2vec |  |  |  |  | 1.00 |

Above p values are based on the MCC and AUC. Color thresholds are 0.05 (blue), 0.07 (white), and 0.3 (red). The values between the thresholds are expressed in the middle color such as light blue for 0.06, which is between blue and white.

Table S5: Overview of *p* values obtained with Students T test on protein features

|  | AA_seq | ProtVec | AA+ ProtVec |
| --- | --- | --- | --- |
| AA_seq | 1.00 | 0.08 | 0.03 |
| ProtVec |  | 1.00 | 0.02 |
| AA+ ProtVec |  |  | 1.00 |

Above p values are based on the MCC and AUC. Color thresholds are 0.05 (blue), 0.07 (white), and 0.3 (red). The values between the thresholds are expressed in the middle color such as light red for 0.08, which is between red and white.

Table S6: Overview of *p* values obtained with Fisher F test on protein features

| protein | AA_seq | ProtVec | AA+ ProtVec |
| --- | --- | --- | --- |
| AA_seq | 1.00 | 0.02 | 0.00 |
| ProtVec |  | 1.00 | 0.00 |
| AA+ ProtVec |  |  | 1.00 |

Above p values are based on the MCC and AUC. Color thresholds are 0.05 (blue), 0.07 (white), and 0.3 (red).

Table S7: Overview of *p* values obtained with Students T test on all model

| models | SC2 | SC1 | MC1 | MC4 | MC5 | MC8 | SC5 | MC6 | SC6 | MC7 | SC3 | MC3 | MC9 | SC4 | MC2 |
| --- | --- | --- | --- | --- | --- | --- | --- | --- | --- | --- | --- | --- | --- | --- | --- |
| SC2 | 1.00 | 0.02 | 0.02 | 0.03 | 0.01 | 0.02 | 0.01 | 0.00 | 0.01 | 0.01 | 0.00 | 0.00 | 0.00 | 0.00 | 0.00 |
| SC1 |  | 1.00 | 0.00 | 0.05 | 0.03 | 0.04 | 0.01 | 0.00 | 0.00 | 0.00 | 0.00 | 0.01 | 0.00 | 0.01 | 0.01 |
| MC1 |  |  | 1.00 | 0.05 | 0.03 | 0.04 | 0.01 | 0.00 | 0.00 | 0.00 | 0.00 | 0.01 | 0.00 | 0.01 | 0.01 |
| MC4 |  |  |  | 1.00 | 0.43 | 0.01 | 0.13 | 0.08 | 0.07 | 0.07 | 0.06 | 0.03 | 0.05 | 0.03 | 0.03 |
| MC5 |  |  |  |  | 1.00 | 0.11 | 0.10 | 0.05 | 0.05 | 0.05 | 0.04 | 0.01 | 0.03 | 0.01 | 0.02 |
| MC8 |  |  |  |  |  | 1.00 | 0.20 | 0.12 | 0.08 | 0.09 | 0.07 | 0.04 | 0.06 | 0.03 | 0.03 |
| SC5 |  |  |  |  |  |  | 1.00 | 0.23 | 0.04 | 0.04 | 0.05 | 0.09 | 0.04 | 0.06 | 0.03 |
| MC6 |  |  |  |  |  |  |  | 1.00 | 0.03 | 0.03 | 0.01 | 0.05 | 0.00 | 0.04 | 0.01 |
| SC6 |  |  |  |  |  |  |  |  | 1.00 | 0.50 | 0.07 | 0.22 | 0.05 | 0.09 | 0.03 |
| MC7 |  |  |  |  |  |  |  |  |  | 1.00 | 0.10 | 0.23 | 0.06 | 0.10 | 0.03 |
| SC3 |  |  |  |  |  |  |  |  |  |  | 1.00 | 0.34 | 0.03 | 0.10 | 0.02 |
| MC3 |  |  |  |  |  |  |  |  |  |  |  | 1.00 | 0.75 | 0.01 | 0.02 |
| MC9 |  |  |  |  |  |  |  |  |  |  |  |  | 1.00 | 0.13 | 0.02 |
| SC4 |  |  |  |  |  |  |  |  |  |  |  |  |  | 1.00 | 0.04 |
| MC2 |  |  |  |  |  |  |  |  |  |  |  |  |  |  | 1.00 |

Above p values are based on the MCC and AUC. Color thresholds are 0.05 (blue), 0.07 (white), and 0.3 (red). The values between the thresholds are expressed in the middle color such as light red for 0.1, which is between red and white.

Table S8: Overview of *p* values obtained with Fisher F test on all model

|  | SC2 | SC1 | MC1 | MC4 | MC5 | MC8 | SC5 | MC6 | SC6 | MC7 | SC3 | MC3 | MC9 | SC4 | MC2 |
| --- | --- | --- | --- | --- | --- | --- | --- | --- | --- | --- | --- | --- | --- | --- | --- |
| SC2 | 1.00 | 0.00 | 0.00 | 0.00 | 0.00 | 0.00 | 0.00 | 0.00 | 0.00 | 0.00 | 0.00 | 0.00 | 0.00 | 0.00 | 0.00 |
| SC1 |  | 1.00 | 0.18 | 0.00 | 0.00 | 0.00 | 0.00 | 0.00 | 0.00 | 0.00 | 0.00 | 0.00 | 0.00 | 0.00 | 0.00 |
| MC1 |  |  | 1.00 | 0.00 | 0.00 | 0.00 | 0.00 | 0.00 | 0.00 | 0.00 | 0.00 | 0.00 | 0.00 | 0.00 | 0.00 |
| MC4 |  |  |  | 1.00 | 0.65 | 0.15 | 0.02 | 0.01 | 0.01 | 0.01 | 0.01 | 0.00 | 0.00 | 0.00 | 0.00 |
| MC5 |  |  |  |  | 1.00 | 0.15 | 0.01 | 0.00 | 0.00 | 0.00 | 0.00 | 0.00 | 0.00 | 0.00 | 0.00 |
| MC8 |  |  |  |  |  | 1.00 | 0.05 | 0.02 | 0.01 | 0.01 | 0.01 | 0.01 | 0.01 | 0.00 | 0.00 |
| SC5 |  |  |  |  |  |  | 1.00 | 0.23 | 0.02 | 0.03 | 0.02 | 0.01 | 0.01 | 0.01 | 0.00 |
| MC6 |  |  |  |  |  |  |  | 1.00 | 0.01 | 0.02 | 0.01 | 0.00 | 0.00 | 0.00 | 0.00 |
| SC6 |  |  |  |  |  |  |  |  | 1.00 | 0.95 | 0.21 | 0.06 | 0.05 | 0.01 | 0.00 |
| MC7 |  |  |  |  |  |  |  |  |  | 1.00 | 0.24 | 0.07 | 0.06 | 0.01 | 0.00 |
| SC3 |  |  |  |  |  |  |  |  |  |  | 1.00 | 0.14 | 0.09 | 0.01 | 0.00 |
| MC3 |  |  |  |  |  |  |  |  |  |  |  | 1.00 | 0.61 | 0.02 | 0.00 |
| MC9 |  |  |  |  |  |  |  |  |  |  |  |  | 1.00 | 0.02 | 0.00 |
| SC4 |  |  |  |  |  |  |  |  |  |  |  |  |  | 1.00 | 0.00 |
| MC2 |  |  |  |  |  |  |  |  |  |  |  |  |  |  | 1.00 |

Above p values are based on the MCC and AUC. Color thresholds are 0.05 (blue), 0.07 (white), and 0.3 (red). The values between the thresholds are expressed in the middle color such as light red for 0.14, which is between red and white.

Table S9: Overview of convergence speed for different thresholds.

| Threshold | model | Time(min) | epoch |
| --- | --- | --- | --- |
| 95% | MC9 | **5** | **9** |
|  | MC2 | 18 | 48 |
|  | SC4 | 26 | 95 |
|  | SC1 | 30 | 60 |
| 98% | MC9 | **11** | **18** |
|  | MC2 | 41 | 113 |
|  | SC4 | 55 | 201 |
|  | SC1 | 50 | 102 |
| 99% | MC9 | **30** | **53** |
|  | MC2 | 55 | 147 |
|  | SC4 | 86 | 316 |
|  | SC1 | 105 | 213 |

MC9 consistently demonstrated outstanding performance in convergence speed. MC9 utilizes both multi-channel for compounds and proteins.

Table S10: Overview of p values obtained with Students T test on finetuned models for each pretrained epochs

| Epoch(i) | Pretrained model | PM_i (PRC) | PM_i (MCC) | Baseline:PM_0 (PRC) | Baseline:PM_0 (MCC) | p value |
| --- | --- | --- | --- | --- | --- | --- |
| 0 | PM_0 | 0.481 | 0.430 | 0.481 | 0.430 | 0.64 |
| 5 | PM_5 | 0.482 | 0.434 | 0.481 | 0.430 | 0.33 |
| 10 | PM_10 | 0.486 | 0.436 | 0.481 | 0.430 | 0.05 |
| 15 | PM_15 | 0.490 | 0.437 | 0.481 | 0.430 | 0.08 |
| 20 | PM_20 | 0.494 | 0.440 | 0.481 | 0.430 | 0.07 |
| 25 | PM_25 | 0.494 | 0.440 | 0.481 | 0.430 | 0.06 |
| 30 | PM_30 | 0.496 | 0.446 | 0.481 | 0.430 | 0.01 |
| 35 | PM_35 | 0.496 | 0.444 | 0.481 | 0.430 | 0.03 |
| 40 | PM_40 | 0.498 | 0.446 | 0.481 | 0.430 | 0.02 |
| 45 | PM_45 | 0.498 | 0.446 | 0.481 | 0.430 | 0.02 |
| 50 | PM_50 | 0.498 | 0.447 | 0.481 | 0.430 | 0.00 |
| 55 | PM_55 | 0.500 | 0.450 | 0.481 | 0.430 | 0.02 |
| 60 | PM_60 | 0.499 | 0.447 | 0.481 | 0.430 | 0.02 |
| 65 | PM_65 | 0.499 | 0.447 | 0.481 | 0.430 | 0.01 |
| 70 | PM_70 | 0.499 | 0.446 | 0.481 | 0.430 | 0.03 |
| 75 | PM_75 | 0.499 | 0.447 | 0.481 | 0.430 | 0.02 |
| 80 | PM_80 | 0.496 | 0.446 | 0.481 | 0.430 | 0.03 |
| 85 | PM_85 | 0.495 | 0.446 | 0.481 | 0.430 | 0.053 |
| 90 | PM_90 | 0.495 | 0.445 | 0.481 | 0.430 | 0.01 |
| 95 | PM_95 | 0.496 | 0.444 | 0.481 | 0.430 | 0.01 |
| 100 | PM_100 | 0.495 | 0.443 | 0.481 | 0.430 | 0.01 |
| 105 | PM_105 | 0.492 | 0.441 | 0.481 | 0.430 | 0.01 |
| 110 | PM_110 | 0.492 | 0.441 | 0.481 | 0.430 | 0.01 |
| 115 | PM_115 | 0.491 | 0.442 | 0.481 | 0.430 | 0.07 |
| 120 | PM_120 | 0.492 | 0.442 | 0.481 | 0.430 | 0.02 |
| 125 | PM_125 | 0.492 | 0.441 | 0.481 | 0.430 | 0.03 |
| 130 | PM_130 | 0.491 | 0.439 | 0.481 | 0.430 | 0.01 |
| 135 | PM_135 | 0.489 | 0.438 | 0.481 | 0.430 | 0.01 |

Above value is the raw data of Figure 6 in the main manuscript. p value is obtained on a paired t test. Each PM_i was finetuned for every 5 epochs.
